# A highly sensitive terbium–keton complex-based fluorescent immunoassay for detection of methicillin-resistant *Staphylococcus aureus* (MRSA) antigen in milk, farmer hand swabs, and cattle drinking water

**DOI:** 10.1039/d6ra05840j

**Published:** 2026-09-14

**Authors:** Norhan M. Ali, Shereen S. Maghraby, Nour Eddine M. Ghoneimy, Youssef M. Hassan, Menna H. Mustafa, Ahmed O. Youssef, Wafaa H. Abdel-Ghaffar, Hesham G. Afify, Ekram H. Mohamed, Alaa R. I. Morsy, Ghada M. Elsadek, Mohamed S. Mohy Eldin, Mona N. Abou-Omar, W. A. Abdelkawy, Samir A. Nassif, Mohamed S. Attia

**Affiliations:** a Chemistry Department, Faculty of Science, Ain Shams University Abbassia Cairo 11566 Egypt; b Zoology Department, Faculty of Science, Ain Shams University Abbassia Cairo 11566 Egypt; c Medical Research Laboratories, Ministry of Civil Aviation Cairo Egypt; d Analytical Pharmaceutical Chemistry, Faculty of Pharmacy, British University in Egypt Cairo Egypt; e Central Laboratory for Evaluation of Veterinary Biologics (CLEVB), Agriculture Research Center (ARC) Cairo Egypt; f Polymer Materials Research Department, Advanced Technology and New Materials Research Institute (ATNMRI), City of Scientific Research and Technological Applications (SRTA-City) New Borg El-Arab City 21934 Alexandria Egypt; g Chemistry Department, Faculty of Science, Sadat University Menoufia Egypt; h Soil Department, Faculty of Agriculture, Cairo University Giza Egypt; i Chemistry Department, College of Science, Imam Mohammad Ibn Saud Islamic University (IMSIU) Riyadh 11623 Saudi Arabia mgaber@imamu.edu.sa

## Abstract

This study presents the development and validation of a novel fluorescence-based immunoassay for the rapid and sensitive detection of methicillin-resistant *Staphylococcus aureus* (MRSA) antigen using a terbium–keton complex (Tb-2,keton) as the luminescent probe. The sensing platform operates through fluorescence enhancement upon specific interaction between the MRSA antigen and its corresponding antibody. Comprehensive spectroscopic characterization, including UV-vis absorption and fluorescence emission spectroscopy, was employed to investigate the optical properties and sensing mechanism. The Tb-2,keton complex exhibited characteristic terbium emission peaks, with the most intense signal observed at approximately 545 nm. Upon sequential addition of MRSA antigen and anti-MRSA antibody, significant fluorescence enhancement was observed, indicating successful immunocomplex formation. The biosensing system demonstrated concentration-dependent fluorescence responses, with the emission intensity increasing progressively with increasing antigen concentrations. The proposed method offers advantages of simplicity, rapid response (15 minutes), and high sensitivity for MRSA antigen detection. This work provides a foundation for developing terbium-based fluorescent immunoassays for pathogenic bacteria detection in clinical and food safety applications.

## Abstract 2

Development and validation of a novel fluorescence immunoassay for rapid and sensitive detection of MRSA antigen using a terbium–keton complex (Tb-2,keton) as the luminescent probe.

## Graphical abstract

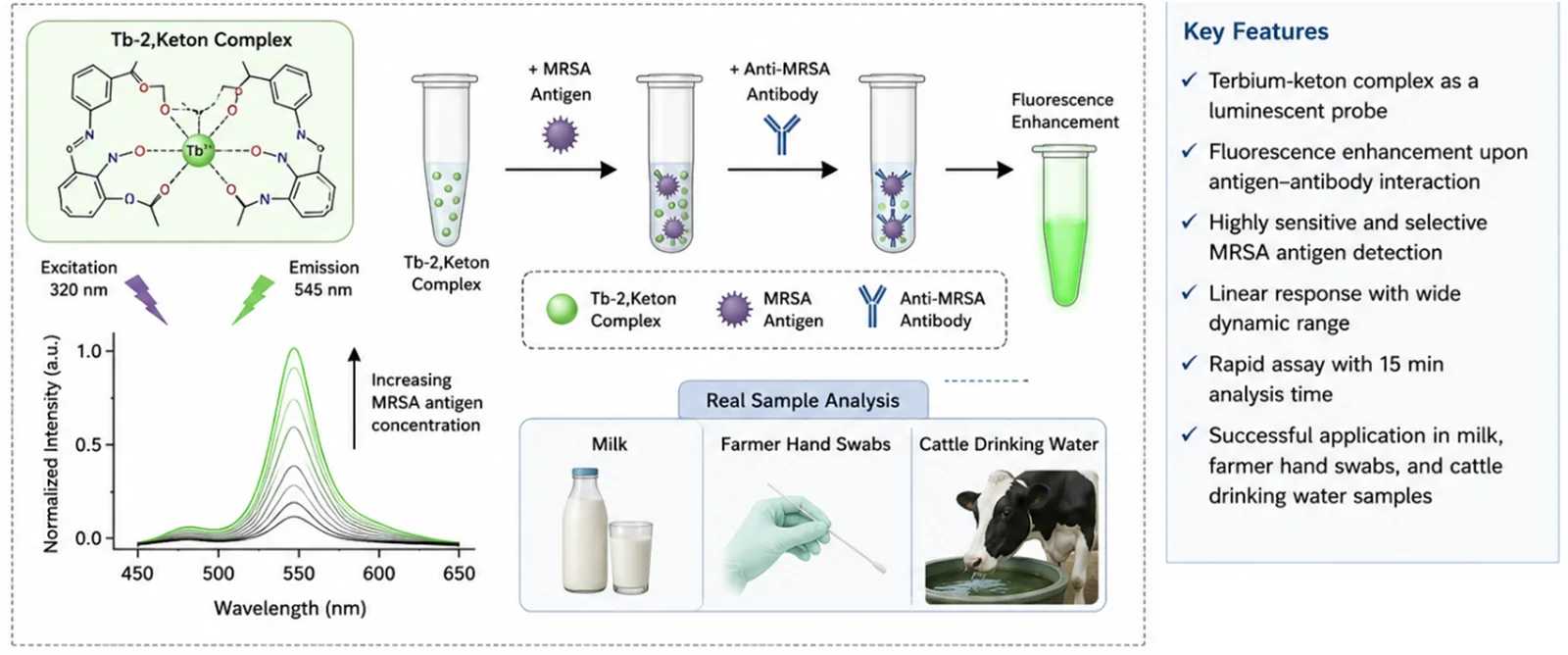

## 1 Introduction

Methicillin-resistant *Staphylococcus aureus* (MRSA) represents one of the most significant public health challenges of the 21st century, causing a wide spectrum of infections ranging from minor skin lesions to life-threatening conditions such as bacteremia, pneumonia, and endocarditis.^1,2^ The emergence and global spread of MRSA, including livestock-associated strains (LA-MRSA), have transformed this pathogen from a hospital-acquired concern to a community and agricultural threat.^3,4^ The contamination of food products, particularly dairy items, and the colonization of food-producing animals and farm workers create complex transmission networks that challenge conventional infection control measures.^5,6^

The molecular basis of methicillin resistance lies in the acquisition of the *mecA* gene, which encodes penicillin-binding protein 2a (PBP2a)—a transpeptidase with low affinity for β-lactam antibiotics.^7,8^ This altered penicillin-binding protein enables bacterial cell wall synthesis to continue in the presence of methicillin and other β-lactam drugs, rendering standard antibiotic therapies ineffective.^9^ The clinical and economic burdens associated with MRSA infections necessitate the development of rapid, sensitive, and specific detection methods that can facilitate timely intervention and surveillance.

Conventional MRSA detection methodologies rely on culture-based techniques followed by antibiotic susceptibility testing and molecular confirmation *via* polymerase chain reaction (PCR) targeting the *mecA* gene.^10,11^ While these methods remain the gold standard due to their high specificity and sensitivity, they are inherently time-consuming, typically requiring 24–72 hours to obtain definitive results.^12^ This temporal gap between sample collection and diagnosis represents a critical limitation in situations requiring immediate clinical decisions or rapid screening in food production environments. Furthermore, these methods demand specialized laboratory infrastructure, trained personnel, and multiple procedural steps, rendering them impractical for point-of-care or on-site applications.^13^

Immunoassay-based methods, particularly enzyme-linked immunosorbent assay (ELISA), have emerged as alternatives offering improved turnaround times (2–4 hours) while maintaining reasonable specificity through antibody-antigen recognition.^14^ However, conventional ELISA still involves multiple incubation and washing steps, requires plate readers, and exhibits detection limits that may not be sufficient for early-stage infection or low-level contamination scenarios.^15^ The limitations of existing methods have driven intensive research into novel detection platforms. Among emerging approaches, lanthanide-based luminescent probes have attracted considerable attention due to their unique photophysical properties.^16,17^ Terbium(iii) complexes offer distinct advantages for bioanalytical applications, including large Stokes shifts (>150 nm), long luminescence lifetimes (microseconds to milliseconds), sharp emission bands, and excellent resistance to photobleaching.^18,19^ These characteristics enable time-resolved fluorescence measurements that effectively eliminate background autofluorescence from biological matrices, significantly enhancing signal-to-noise ratios and detection sensitivity.^20^

The novelty of our work lies in four key aspects: (1) the use of 2-hydroxy-4-methoxybenzophenone (keton ligand) as the antenna chromophore for terbium sensitization—a ligand system not previously reported for MRSA detection; (2) a unique sequential dual-enhancement mechanism where both antigen and antibody addition produce fluorescence enhancement, providing inherent signal amplification; (3) a simple ‘mix-and-measure’ protocol requiring no complex labeling or washing steps; and (4) rapid detection in complex matrices (milk, swabs, water) within 15 minutes. These features distinguish our Tb-2,keton-based immunoassay from previously reported lanthanide-based biosensors and MRSA detection methods.

To date, lanthanide-based immunoassays for pathogen detection have largely relied on time-resolved fluorescence (TRF) using commercially available lanthanide chelates (*e.g.*, Europium-labeled antibodies in DELFIA® systems). While these methods offer excellent sensitivity, they typically require multiple washing steps, labeling of antibodies, and relatively long assay times (1–2 hours). Our approach differs fundamentally in that (i) the terbium complex itself serves as the signal transducer without covalent labeling of antibodies, (ii) both antigen and antibody produce fluorescence enhancement, and (iii) the assay is completed in 15 minutes with a simple mixing protocol. The keton ligand, which we introduce as a novel antenna for terbium sensitization in this context, offers advantages including strong absorption at 320 nm, efficient intersystem crossing, and appropriate triplet state energy level (≈22 500 cm^−1^) that closely matches the ^5^D_4_ accepting level of Tb^3+^ (≈20 500 cm^−1^), enabling efficient energy transfer.^21–25^

Immunoassays integrating lanthanide complexes with specific antibodies combine the sensitivity of fluorescence detection with the selectivity of immunological recognition.^26^ In such platforms, the formation of antigen–antibody complexes can modulate the luminescence of the terbium probe through various mechanisms. The resulting fluorescence changes provide a direct, quantifiable signal proportional to analyte concentration.

The proposed method constitutes a homogeneous fluorescent immunoassay rather than a constructed physical sensor device. The term “immunosensor” is used in the biosensor literature to describe molecular recognition elements that transduce binding events into measurable signals, even when using standard laboratory instrumentation. Throughout this study the using of “immunoassay” and “fluorescent probe” to accurately describe our method while acknowledging its potential for future integration into sensor devices.^27,28^

This study introduces a novel fluorescent immunoassay for MRSA antigen detection based on a terbium–keton complex (Tb-2,keton). We systematically investigate the fluorescence properties of the Tb-2,keton probe and its response to sequential addition of MRSA antigen and anti-MRSA antibody. The concentration-dependent fluorescence enhancement observed upon immunocomplex formation provides the basis for quantitative antigen detection. Through detailed spectroscopic analysis, we elucidate the sensing mechanism and demonstrate the potential of this platform for rapid, sensitive MRSA detection. This work contributes to the growing field of lanthanide-based bioanalytical methods and offers a promising approach for addressing the critical need for improved MRSA surveillance tools in clinical and food safety applications.

## 2 Materials and methods

### 2.1. Reagents and chemicals

All chemicals used in this study were of analytical grade and used without further purification unless otherwise specified. Terbium(iii) nitrate pentahydrate (Tb(NO_3_)_3_·5H_2_O, 99.9% trace metals basis) was procured from Sigma-Aldrich (St. Louis, MO, USA). The keton ligand (2-hydroxy-4-methoxybenzophenone, ≥98%) was obtained from Alfa Aesar (Haverhill, MA, USA). Absolute ethanol (≥99.8%) and dimethyl sulfoxide (DMSO, ≥99.9%) were purchased from Merck (Darmstadt, Germany). HPLC-grade acetonitrile was sourced from Fisher Scientific (Waltham, MA, USA). Deionized water (resistivity 18.2 MΩ cm) was used throughout the experiments and was produced by a Milli-Q water purification system (Merck Millipore, Burlington, MA, USA).

MRSA antigen (0.5 mg mL^−1^, Cat. No. MBS190480) and monoclonal anti-MRSA antibody (0.5 mg mL^−1^, Cat. No. MBS312552) were purchased from MyBioSource (San Diego, CA, USA). The molecular weight of the MRSA antigen is ∼35 kDa as specified by the manufacturer. Phosphate-buffered saline (PBS) tablets (pH 7.4) were obtained from Sigma-Aldrich and used to prepare 10 mM PBS solution. All stock solutions were prepared freshly before use and stored at 4 °C in the dark when not in use.

### 2.2. Instrumentation

UV-visible absorption spectra were recorded using a double-beam spectrophotometer (Shimadzu UV-2600, Kyoto, Japan) equipped with quartz cuvettes of 1.0 cm path length. The instrument was operated with a scan speed of 200 nm min^−1^ and a spectral bandwidth of 2.0 nm over the wavelength range of 250–500 nm. Baseline correction was performed using appropriate blank solutions prior to each measurement. The molar absorptivity of the Tb-2,keton complex at 320 nm was calculated from the absorption spectra using the Beer–Lambert law.

Fluorescence emission spectra were measured using an FS5 spectrofluorometer (Edinburgh Instruments, Livingston, UK) equipped with a xenon arc lamp as the excitation source and a photomultiplier tube detector. All measurements were performed at room temperature (25 ± 1 °C) using quartz cuvettes with 1.0 cm path length. Excitation and emission slit widths were set to 5.0 nm. The instrument was operated in steady-state mode with a dwell time of 0.1 s per data point. Fluorescence emission spectra were recorded from 450 nm to 650 nm upon excitation at the ligand absorption maximum (320 nm), with the most intense emission peak monitored at 545 nm corresponding to the ^5^D_4_ → ^7^F_5_ transition of terbium(iii).

pH measurements were conducted using a SevenCompact pH meter (Mettler Toledo, Columbus, OH, USA) calibrated with standard buffer solutions (pH 4.01, 7.00, and 10.01) prior to each use. All glassware was thoroughly cleaned with dilute nitric acid, rinsed repeatedly with deionized water, and dried before use to prevent contamination that could affect fluorescence measurements.

### 2.3. Preparation of terbium–keton complex (Tb-2,keton)

The terbium–keton complex was prepared by a simple mixing method in ethanol solution based on the optimized ligand-to-metal ratio of 2 : 1, which was determined from preliminary experiments to yield maximum fluorescence intensity.

A stock solution of Tb(NO_3_)_3_·5H_2_O (1.0 × 10^−3^ M) was prepared by dissolving the appropriate amount in absolute ethanol. The keton ligand stock solution (1.0 × 10^−3^ M) was prepared by dissolving 2-hydroxy-4-methoxybenzophenone in absolute ethanol.

For the preparation of the Tb-2,keton complex, 2.0 mL of Tb^3+^ stock solution (1.0 × 10^−3^ M) was mixed with 4.0 mL of keton ligand stock solution (1.0 × 10^−3^ M) in a 10 mL volumetric flask to achieve a final Tb^3+^ concentration of 2.0 × 10^−4^ M and ligand concentration of 4.0 × 10^−4^ M (ligand-to-metal ratio of 2 : 1). The mixture was diluted to volume with absolute ethanol and allowed to equilibrate for 30 minutes at room temperature with gentle shaking to ensure complete complex formation. The formation of the Tb-2,keton complex was confirmed by monitoring the characteristic terbium emission peaks at 490, 545, 585, and 620 nm upon excitation at the ligand absorption wavelength (320 nm). The molar absorptivity of the complex at 320 nm was determined to be 1.85 × 10^4^ M^−1^ cm^−1^, confirming efficient light absorption by the antenna ligand.

### 2.4. Preparation of MRSA antigen and antibody solutions

MRSA antigen stock solution (0.5 mg mL^−1^) was used as received and stored at 4 °C. Working solutions of MRSA antigen at various concentrations (0.05–150 µg mL^−1^) were prepared by appropriate serial dilution of the stock solution with 10 mM PBS buffer (pH 7.4). For experiments involving high antigen concentration (150 µL addition), the stock solution was used without dilution and the final concentration in the cuvette after adding 150 µL of 0.5 mg mL^−1^ stock to 3.0 mL is 23.8 µg mL^−1^ (0.68 µM based on 35 kDa MW).

Anti-MRSA monoclonal antibody stock solution (0.5 mg mL^−1^) was similarly stored at 4 °C. Anti-MRSA antibody is IgG with MW ∼150 kDa. Working solutions of the antibody at various concentrations were prepared by serial dilution of the stock solution with PBS buffer (pH 7.4) to achieve the desired concentrations for titration experiments. All diluted solutions were prepared freshly on the day of experiments and kept on ice during use to maintain antibody activity.

### 2.5. Spectroscopic measurements and sensing protocol

A 3.0 mL aliquot of the prepared Tb-2,keton complex solution (2.0 × 10^−4^ mol L^−1^ Tb^3+^, 4.0 × 10^−4^ mol L^−1^ keton ligand in 10 mmol PBS buffer, pH 7.4, containing 5% ethanol as co-solvent) was placed in a quartz cuvette. The ethanol content was minimized to 5% (v/v) to maintain complex solubility while preserving native protein structure and antibody–antigen binding activity.

To investigate the concentration-dependent response to MRSA antigen, increasing volumes (10–150 µL) of MRSA antigen stock solution (0.5 mg mL^−1^ in PBS, pH 7.4) were added sequentially to the Tb-2,keton complex solution. After each addition, the solution was mixed thoroughly and incubated for 5 minutes at room temperature to facilitate antigen binding to the terbium complex. Following antigen titration, aliquots of anti-MRSA monoclonal antibody solution (0.5 mg mL^−1^ in PBS, pH 7.4) were added to induce immunocomplex formation. The final ethanol concentration remained below 6% (v/v) throughout all measurements, ensuring preservation of protein structure and antibody-antigen binding activity.

### 2.6. UV-vis absorption measurements

UV-vis absorption spectra of the Tb-2-keton complex was recorded before and after the addition of MRSA antigen and antibody to investigate ground-state interactions that might contribute to the observed fluorescence changes. Six distinct spectra were acquired over the wavelength range of 250–500 nm using ethanol as the blank reference: Tb-2,keton complex alone (2.0 × 10^−4^ M), MRSA antigen alone (50 µg mL^−1^ in PBS), Anti-MRSA antibody alone (50 µg mL^−1^ in PBS), Tb-2,keton complex + MRSA antigen (150 µL), Tb-2,keton complex + anti-MRSA antibody (10 µL), and Tb-2,keton complex + MRSA antigen (150 µL) + anti-MRSA antibody (10 µL). The absorption characteristics, including peak positions, intensities, and spectral broadening, were analyzed to identify any spectral shifts or new band formation upon addition of the biological components. The molar absorptivity of the Tb-2,keton complex at 320 nm was calculated using the Beer–Lambert law.

### 2.7. Data analysis

All spectroscopic data were processed using OriginPro software (version 2021, OriginLab Corporation, Northampton, MA, USA). Fluorescence intensities at the maximum emission wavelength (545 nm) were extracted from each spectrum and used for quantitative analysis. The fluorescence enhancement efficiency was calculated using the equation:Enhancement (%) = [(*I* − *I*_0_)/*I*_0_] × 100where *I*_0_ is the fluorescence intensity of the Tb-2,keton complex alone and *I* is the fluorescence intensity after addition of antigen or antibody.

For calibration curve construction, the fluorescence intensity ratio (*F*/*F*_0_) at 545 nm was plotted against MRSA antigen concentration (µg mL^−1^). Linear regression analysis was performed to determine the slope, intercept, and correlation coefficient. The limit of detection (LOD) and limit of quantification (LOQ) were calculated according to IUPAC guidelines using the equations:LOD = 3.3 × *σ*/*S*LOQ = 10 × *σ*/*S*where *σ* is the standard deviation of the blank response (ten replicate measurements of the Tb-2,keton complex without antigen) and *S* is the slope of the calibration curve.

All experiments were performed at least three times, and results are expressed as mean ± standard deviation (SD) where applicable.

### 2.8. Analytical validation for MRSA determination in real samples

#### 2.8.1. Sample collection and preparation

The practical utility of the Tb-2,keton-based immunoassay was evaluated by analyzing real samples collected from dairy farms in the Cairo, Giza, and Qalyubia governorates of Egypt. A total of 120 samples were analyzed: 40 raw milk samples, 40 farmer hand swab samples, and 40 cattle drinking water samples.

Raw milk samples (10 mL each) were collected in sterile containers from individual cows and transported to the laboratory at 4 °C. Samples were centrifuged at 10 000 rpm for 15 minutes at 4 °C to separate fat and debris. The clear whey fraction (intermediate layer) was carefully collected using a sterile pipette, filtered through 0.45 µm membrane filters to remove any remaining particulate matter, and diluted 1 : 10 with PBS buffer (pH 7.4) to minimize matrix effects. The diluted samples were stored at 4 °C and analyzed within 24 hours of collection.

Sterile cotton swabs moistened with sterile PBS buffer were used to sample both hands of farm workers immediately after milking operations. The swabs were wiped across the palms, fingers, and interdigital spaces of both hands. Each swab was placed in a sterile 15 mL tube containing 2.0 mL PBS buffer and vortexed vigorously for 2 minutes to elute adherent material. The resulting suspension was filtered through 0.22 µm syringe filters to remove debris and bacterial cells (to comply with biosafety requirements). The filtered eluate was used directly for analysis without dilution to maintain detectable antigen concentrations.

Water samples (100 mL each) were collected from cattle drinking troughs in sterile polypropylene bottles. Samples were transported to the laboratory at 4 °C and processed within 4 hours of collection. Each sample was filtered through 0.22 µm membrane filters to concentrate any bacterial cells and remove particulate matter. The filters were then placed in 2.0 mL PBS buffer and vortexed for 5 minutes to elute adherent material. The resulting suspension was used directly for analysis.

#### 2.8.2. Analysis protocol for real samples

For milk sample analysis, 150 µL of the diluted whey fraction was added to 3.0 mL of Tb-2,keton complex solution (2.0 × 10^−4^ M) in a quartz cuvette. The mixture was incubated for 5 minutes at room temperature, and the fluorescence emission spectrum was recorded at *λ*_ex_ = 320 nm. Subsequently, 10 µL of anti-MRSA monoclonal antibody solution (0.5 mg mL^−1^) was added, followed by another 5 minute incubation and fluorescence measurement. For hand swab samples, 200 µL of the filtered eluate was used directly following the same protocol.

The enhancement ratio (*F*/*F*_0_) was calculated for each sample, where *F*_0_ is the fluorescence intensity of the Tb-2,keton complex alone and *F* is the intensity after sequential addition of sample and antibody. MRSA antigen concentrations in the samples were determined by interpolation from a calibration curve constructed using standard MRSA antigen solutions (0.05–150 µg mL^−1^) prepared in PBS buffer and analyzed under identical conditions.

#### 2.8.3. Reference method

For validation purposes, all samples were also analyzed using the reference method: culture on Oxacillin Resistance Screening Agar Base (ORSAB) followed by PCR confirmation of the *mecA* gene. Briefly, 100 µL of each milk sample (or swab eluate) was spread onto ORSAB plates and incubated at 37 °C for 24–48 hours. Characteristic blue colonies were subcultured and confirmed as *S. aureus* by Gram staining, catalase test, and coagulase test. DNA was extracted from confirmed colonies using a commercial extraction kit, and PCR amplification of the *mecA* gene was performed using specific primers. Samples were considered positive only when both culture and PCR were positive. Quantitative results from the reference method were obtained by serial dilution and colony counting, converted to antigen concentration equivalents based on a predetermined correlation factor (1 CFU ≈ 0.0003 µg antigen, determined experimentally).

#### 2.8.4. Recovery studies

Recovery studies were performed to evaluate matrix effects and method accuracy. Known concentrations of MRSA antigen (low, medium, and high) were spiked into negative milk and hand swab samples (previously confirmed negative by both the proposed method and reference method). For milk samples, spiking concentrations were 5.0, 25.0, and 100.0 µg mL^−1^. For hand swab samples, spiking concentrations were 1.0, 10.0, and 50.0 µg mL^−1^. Spiked samples were analyzed following the same protocol as for real samples, and recoveries were calculated as:Recovery (%) = (Found concentration/Spiked concentration) × 100

Each spiking level was analyzed in five replicates (*n* = 5), and results were expressed as mean ± SD with % RSD.

#### 2.8.5. Selectivity assessment

The selectivity of the Tb-2,keton immunoassay was evaluated by testing its response to potential interfering substances commonly present in milk and swab samples, as well as cross-reactivity with other bacterial species. The following interferents were tested at the indicated concentrations: Bovine serum albumin (100 µg mL^−1^), casein (100 µg mL^−1^), lactose (100 µg mL^−1^), glucose (100 µg mL^−1^), urea (100 µg mL^−1^), whey protein (100 µg mL^−1^), lactoferrin (50 µg mL^−1^). Na^+^ (100 mM NaCl), K^+^ (100 mM KCl), Ca^2+^ (10 mM CaCl_2_), Mg^2+^ (10 mM MgCl_2_). Veterinary drugs: Penicillin (10 µg mL^−1^), tetracycline (10 µg mL^−1^). Other bacteria (heat-killed suspensions, 10^7^ CFU mL^−1^) : *Escherichia coli*, *Salmonella enterica*, *Listeria monocytogenes*, methicillin-sensitive *Staphylococcus aureus* (MSSA), *Staphylococcus epidermidis*, *Streptococcus agalactiae*, *Klebsiella pneumoniae*, *Pseudomonas aeruginosa*. Each interferent was added to the Tb-2,keton complex solution following the same sequential protocol (interferent first, then antibody), and the fluorescence response was recorded. The relative signal was calculated as:Relative signal (%) = [(*F*/*F*_0_ of interferent − 1)/(*F*/*F*_0_ of MRSA − 1)] × 100where *F*/*F*_0_ of MRSA is the response to 1.0 µg mL^−1^ MRSA antigen.

#### 2.8.6. Stability studies

The stability of the Tb-2,keton complex was evaluated by storing the prepared solution under two different conditions: (1) refrigerated at 4 °C in the dark, and (2) at room temperature (25 °C) in the dark. At weekly intervals over a period of 12 weeks, aliquots were withdrawn and tested for their fluorescence response to a standard MRSA antigen solution (50 µg mL^−1^) following the standard protocol. The response was compared to the initial response (week 0) and expressed as percentage of initial response. The physical appearance of the solution (clarity, precipitation) and spectral characteristics (peak positions, shape) were also monitored.

#### 2.8.7. Reproducibility and robustness

##### 2.8.7.1 Inter-batch reproducibility

Three independent batches of Tb-2,keton complex were prepared on different days by the same operator. Each batch was tested for its response to the same panel of MRSA antigen standards (1.0, 5.0, 10.0, 25.0, 50.0, and 100.0 µg mL^−1^) following the standard protocol. The mean response, standard deviation, and % RSD were calculated for each concentration to assess batch-to-batch variability.

The robustness of the method was evaluated by introducing small deliberate variations in experimental parameters and observing the effect on the analytical response for a 50 µg mL^−1^ MRSA antigen standard. The following parameters were varied individually while keeping all other conditions at optimal values: excitation wavelength: 318 nm, 320 nm (optimal), 322 nm, emission wavelength: 543 nm, 545 nm (optimal), 547 nm, pH of PBS buffer: 7.2, 7.4 (optimal), 7.6, incubation time: 4 min, 5 min (optimal), 6 min, temperature: 23 °C, 25 °C (optimal), 27 °C, and Tb-2,keton concentration: 1.8 × 10^−4^ M, 2.0 × 10^−4^ M (optimal), 2.2 × 10^−4^ M. The response under each variation was compared to the optimal conditions, and the percent change was calculated.

#### 2.8.8. Statistical analysis

All statistical analyses were performed using OriginPro 2021 and Microsoft Excel. Results are expressed as mean ± standard deviation (SD) for replicate measurements. The percent relative error (% RE) between the proposed method and reference method was calculated as:% RE = [(Proposed value − Reference value)/Reference value] × 100

The percent relative standard deviation (% RSD) was calculated as:% RSD = (SD/Mean) × 100

For method comparison, correlation analysis was performed between the results obtained by the proposed method and the reference method. A *p*-value < 0.05 was considered statistically significant. ANOVA was used for selectivity data analysis.

## 3 Results and discussion

### 3.1. Spectral characteristics of the Tb-2,keton complex

The photophysical properties of the terbium–keton complex (Tb-2,keton) form the foundation of its application as a fluorescent probe for MRSA detection. Lanthanide complexes exhibit characteristic emission patterns that are highly sensitive to their coordination environment, making them excellent candidates for sensing applications.^29^

**Fig. 1:**
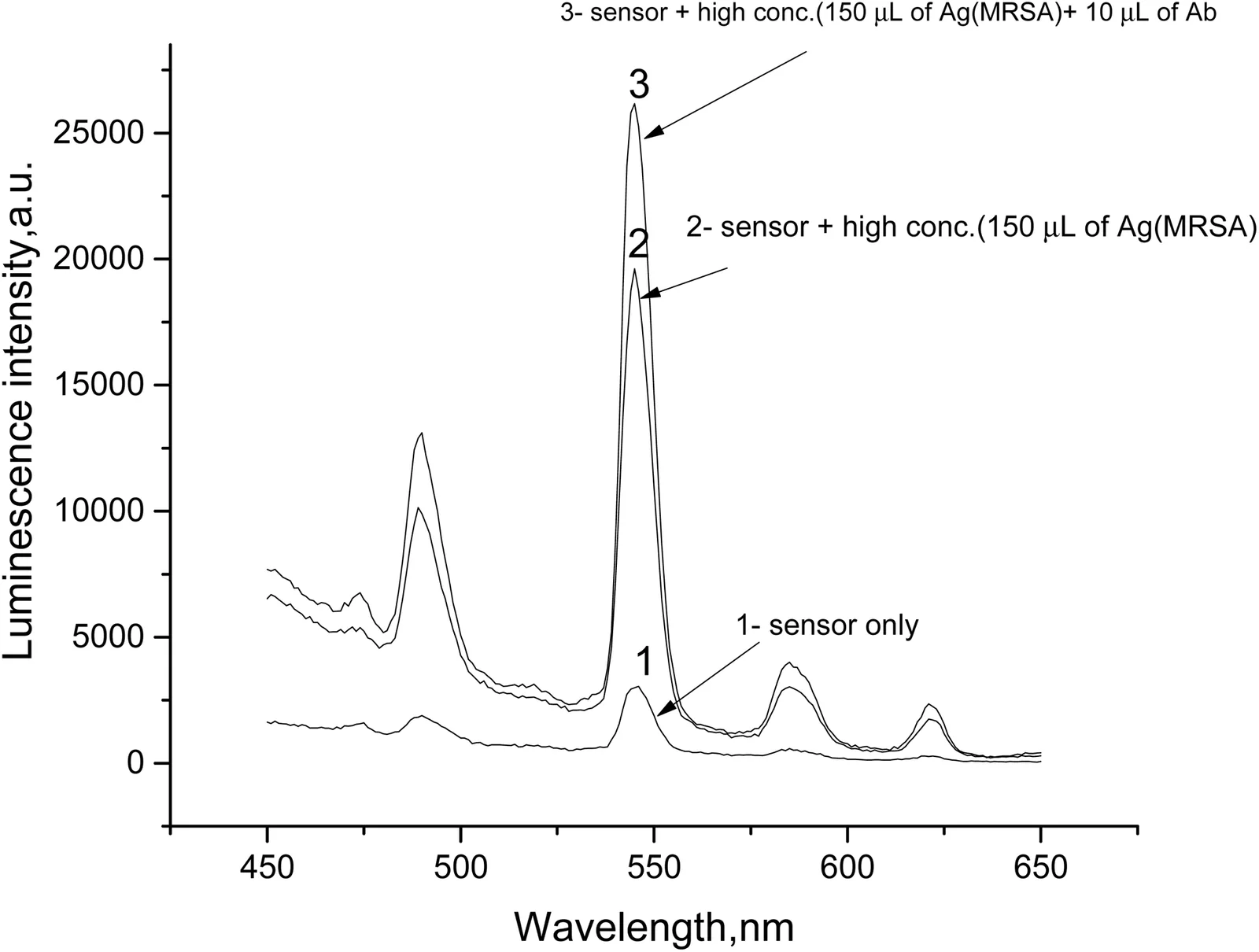
Luminescence spectra of Tb-2–keton complex sensor at *λ*_ex_ = 320 nm: (1) sensor only (Tb-2,keton complex), (2) sensor + 150 µL MRSA antigen (0.5 mg mL^−1^ stock, final ≈23.8 µg mL^−1^), and (3) sensor + 150 µL MRSA antigen + 10 µL anti-MRSA antibody (0.5 mg mL^−1^ stock).

Fig. 1 (Spectrum 1) presents the fluorescence emission spectrum of the Tb-2,keton complex upon excitation at the ligand absorption wavelength (320 nm). The spectrum displays the characteristic narrow emission bands typical of terbium(iii) luminescence, corresponding to transitions from the excited ^5^D_4_ state to various levels of the ^7^F ground state manifold.^30^ The most intense emission peak is observed at approximately 545 nm, corresponding to the ^5^D_4_ → ^7^F_5_ transition, which is responsible for the characteristic green luminescence of terbium complexes. Additional peaks are observed at approximately 490 nm (^5^D_4_ → ^7^F_6_), 585 nm (^5^D_4_ → ^7^F_4_), and 620 nm (^5^D_4_ → ^7^F_3_).

The observation of strong terbium-centered luminescence upon excitation at the ligand absorption wavelength confirms successful energy transfer from the keton ligand to the Tb^3+^ ion—the classic “antenna effect”.^31^ The keton ligand (2-hydroxy-4-methoxybenzophenone) absorbs UV radiation efficiently due to its aromatic structure and conjugated carbonyl group, undergoes intersystem crossing to the triplet state, and subsequently transfers energy to the ^5^D_4_ level of Tb^3+^, from which radiative emission occurs.^32^ The absence of significant residual ligand fluorescence indicates efficient energy transfer, a desirable characteristic for sensing applications as it simplifies the spectral response.

The sharp, well-resolved emission bands are characteristic of terbium complexes and offer several advantages for analytical applications. The narrow bandwidth (typically <10 nm full width at half maximum) minimizes spectral overlap with potential interfering species and enables high-resolution detection.^33^ Furthermore, the large Stokes shift (>200 nm between excitation and emission maxima) eliminates self-quenching and reduces interference from scattered excitation light, both of which are common challenges in fluorescence-based assays using organic fluorophores.^34^

### 3.2. Effect of MRSA antigen on Tb-2,keton fluorescence

The interaction between the Tb-2,keton complex and MRSA antigen was investigated by recording the fluorescence emission spectrum after addition of high-concentration antigen to the complex solution. Fig. 1 (Spectrum 2) shows the emission spectrum of Tb-2,keton following addition of high concentration MRSA antigen.

Comparison of (Fig. 1, spectrum 2) with the baseline spectrum (Fig. 1, spectrum 1) reveals that addition of MRSA antigen alone induces a noticeable change in the fluorescence intensity of the Tb-2,keton complex. The characteristic terbium emission peaks remain at the same wavelengths, indicating that the coordination environment of Tb^3+^ is not fundamentally altered. However, the emission intensity shows a measurable increase compared to the initial complex.

This antigen-induced fluorescence enhancement can be attributed to several possible mechanisms. MRSA antigen, being a proteinaceous material, contains various functional groups (amino, carboxyl, hydroxyl) that can interact with the terbium complex through hydrogen bonding, electrostatic interactions, or coordination to the terbium center.^35^ Such interactions may enhance the efficiency of the antenna effect and energy transfer processes.^36^ Additionally, the antigen may stabilize the excited state of the complex, leading to enhancement of the luminescence quantum yield.^37^

The observation that antigen alone can modulate the fluorescence of the Tb-2,keton complex is significant, as it suggests the possibility of direct antigen detection without the need for secondary recognition elements. However, the selectivity of such direct interactions would likely be limited, as many proteins and biomolecules could similarly interact with the complex. This limitation underscores the importance of incorporating specific biological recognition elements—in this case, the anti-MRSA antibody—to achieve the desired selectivity for MRSA detection.

The core of the immunoassay strategy lies in the specific recognition between MRSA antigen and its corresponding antibody, and the transduction of this recognition event into a measurable fluorescence signal. Fig. 1 (spectrum 3) illustrates the fluorescence emission spectra of the Tb-2,keton complex containing high concentration MRSA antigen upon addition of anti-MRSA antibody.

### 3.3. Concentration-dependent response to MRSA antigen

**Fig. 2:**
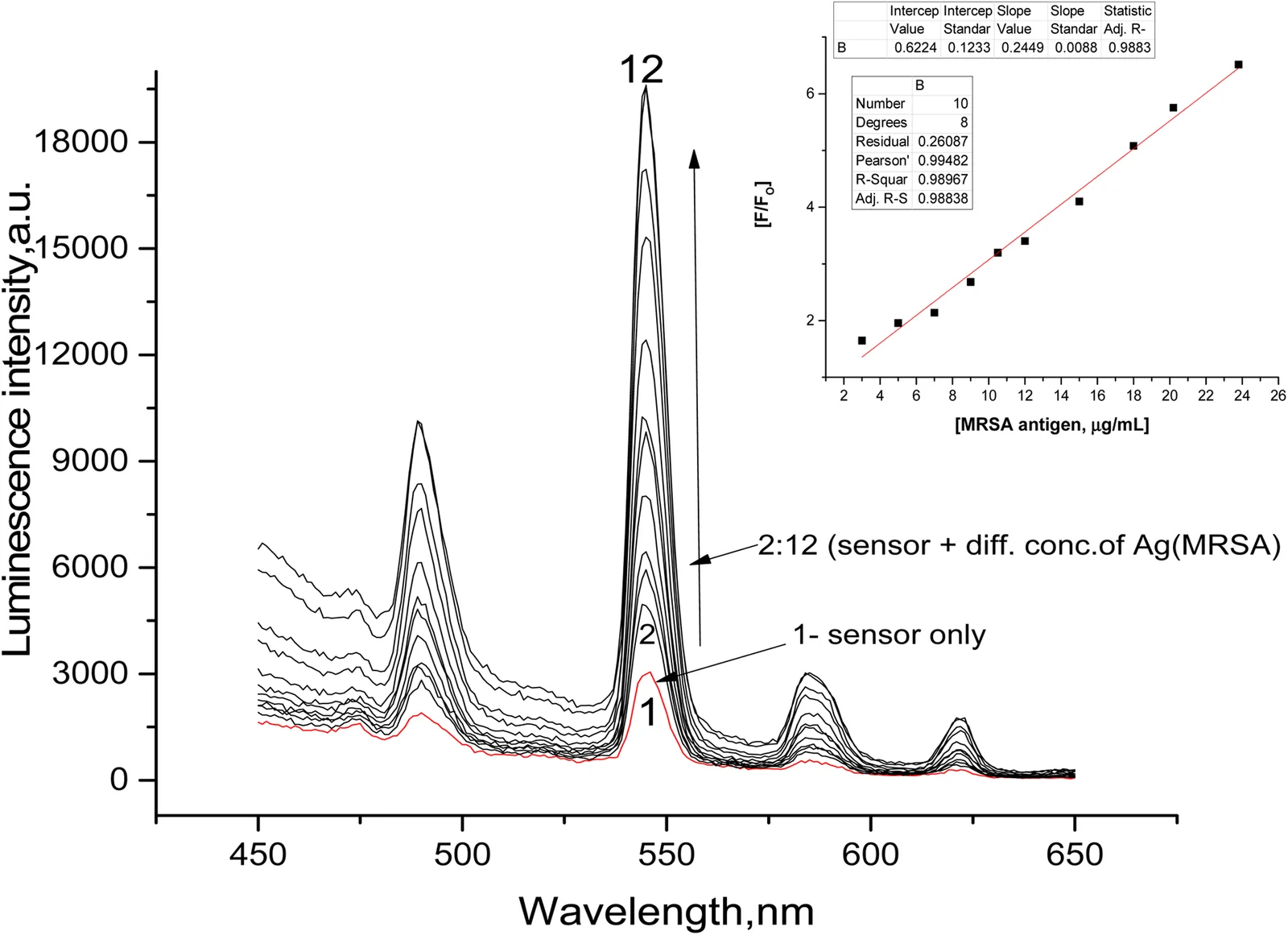
Luminescence spectra of Tb-2–keton complex sensor at *λ*_ex_ = 320 nm: (1) emission spectrum of sensor only, (2–12) sensor + different conc of MRSA antigen. Inset: calibration curve showing linear relationship between *F*/*F*_0_ and MRSA antigen concentration.

Fig. 2 presents the fluorescence emission spectra of the Tb-2,keton immunoassay upon titration with increasing concentrations of MRSA antigen. The spectral series reveals a systematic and progressive enhancement of the characteristic terbium emission bands as the antigen concentration increases. The baseline spectrum (labeled “sensor only”) shows the typical terbium luminescence profile with the dominant ^5^D_4_ → ^7^F_5_ transition at 545 nm, accompanied by the secondary transitions at 490 nm (^5^D_4_ → ^7^F_6_) and approximately 585 nm (^5^D_4_ → ^7^F_4_).

As successive aliquots of MRSA antigen are introduced (2–12 in Fig. 2), the intensity of all emission bands increases proportionally, while the spectral shape remains remarkably consistent. This preservation of spectral profile is critically important as it indicates that the fundamental coordination environment of the terbium ion—the ligand field symmetry and the energy level structure—remains essentially unchanged throughout the titration. If the antigen were displacing ligands or significantly altering the terbium coordination sphere, we would expect to observe spectral shifts, changes in relative peak intensities, or the emergence of new emission bands.^1^ The absence of such changes suggests that the antigen interacts with the complex in a manner that enhances the efficiency of the existing luminescence processes rather than creating an entirely new emitting species. The enhancement continues monotonically until approximately 150 microliters of antigen solution have been added, at which point further additions produce no additional increase in fluorescence intensity. This saturation behavior is clearly evident in the spectral overlay, where the uppermost traces converge to a common maximum intensity. The plateau formation indicates that all available interaction sites on or around the Tb-2,keton complexes have been occupied by antigen molecules, and the system has reached its maximum enhancement capacity.^2^

#### 3.3.1. Mechanisms of antigen-induced fluorescence enhancement

**Fig. 3:**
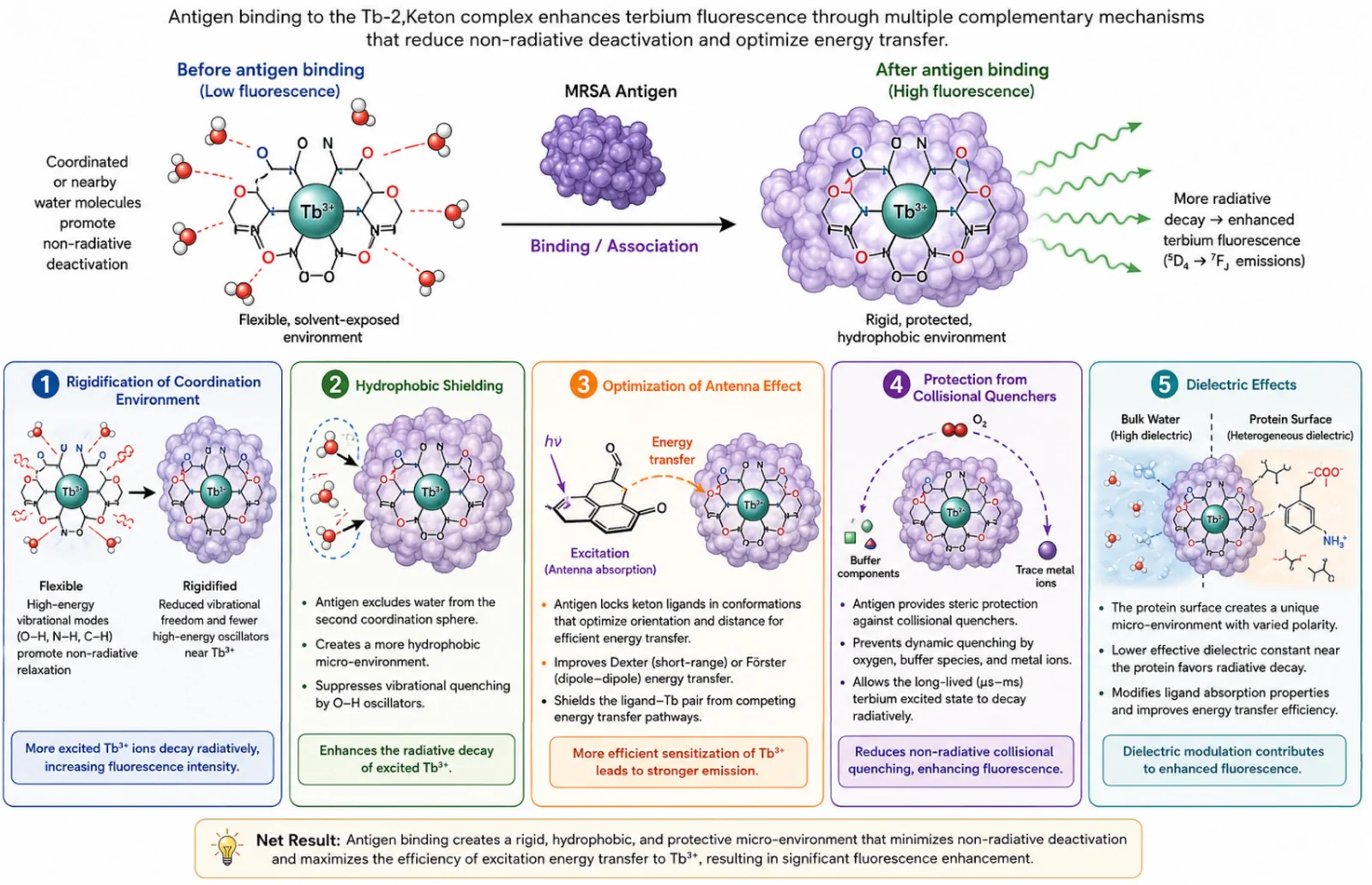
Mechanisms of antigen-induced fluorescence enhancement.

The observed fluorescence enhancement upon antigen addition can be attributed to several complementary mechanisms, each contributing to the increased luminescence output of the terbium probe, Fig. 3. The following complementary hypotheses for the antigen-induced fluorescence enhancement have been proposed:

*Hypothesis 1*: rigidification of the terbium coordination environment. The observed enhancement upon antigen addition may result from rigidification of the terbium coordination environment, which would reduce non-radiative deactivation through coupling with high-energy vibrational modes of O–H, N–H, and C–H oscillators. This hypothesis is supported by the absence of spectral shifts upon antigen addition, which indicates that the coordination sphere is not fundamentally altered, but rather constrained.^3–5^

*Hypothesis 2*: displacement of quenching water molecules. The antigen may displace water molecules from the terbium coordination environment, creating a more hydrophobic local environment that suppresses O–H vibrational quenching. This is consistent with the observed intensity increase without spectral change.^6,7^

*Hypothesis 3*: protection from collisional quenchers. The antigen may provide steric protection against collisional quenchers (dissolved oxygen, buffer components) that could deactivate the excited state. This hypothesis could be tested by comparing Stern–Volmer quenching constants in the presence and absence of antigen.^8^

*Hypothesis 4*: optimization of energy transfer. The antigen may lock the keton ligands into conformations that optimize their orientation for energy transfer to terbium. This is supported by the UV-vis absorption changes upon antigen addition (Fig. 8), which indicate ground-state interactions.^9,10^

#### 3.3.2. Binding isotherm analysis

**Fig. 4:**
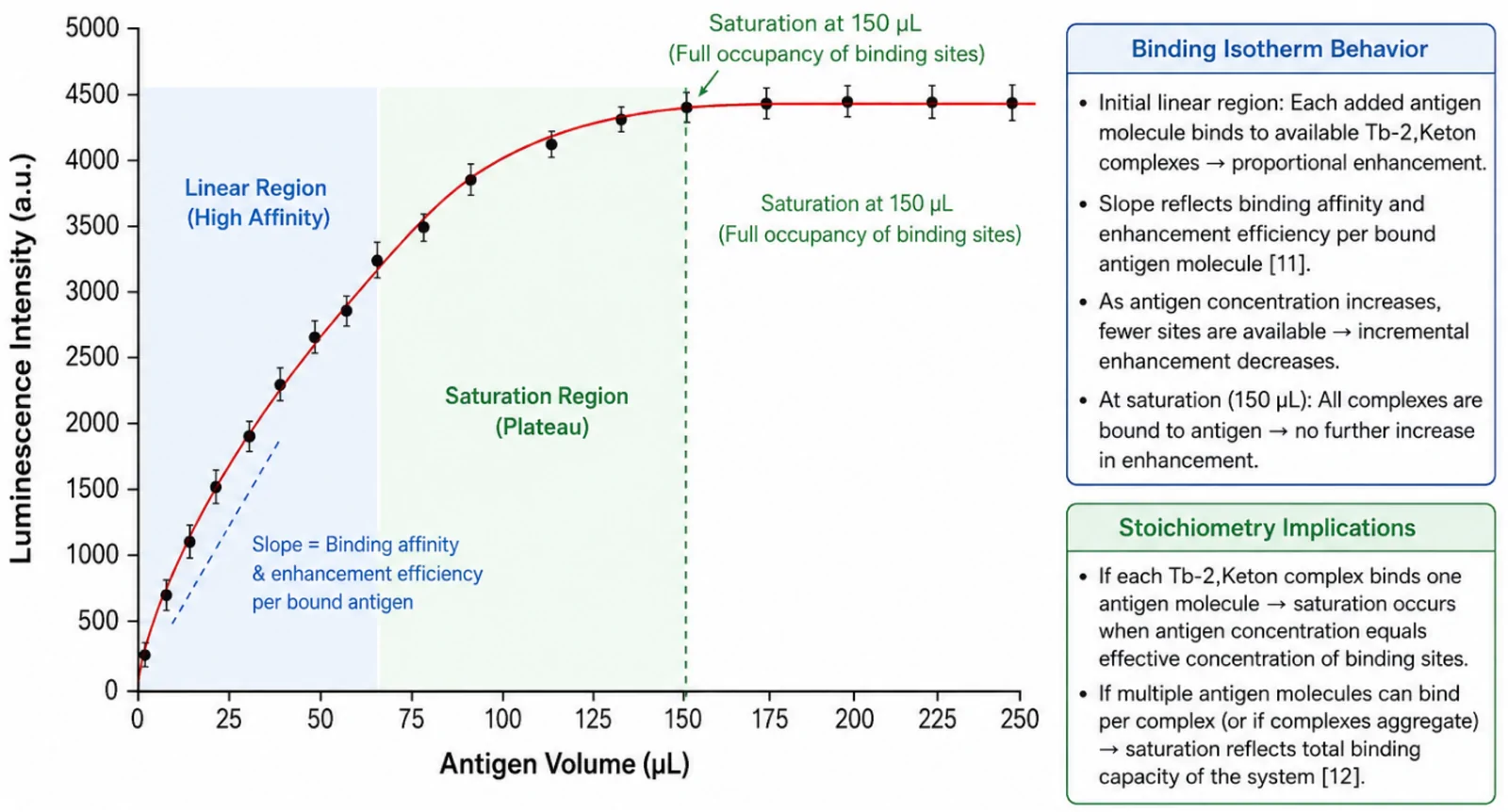
Binding isotherm analysis.

The progressive enhancement with increasing antigen concentration followed by plateau formation at 150 microliters follows classic binding isotherm behavior characteristic of saturable interactions. This pattern provides strong evidence that the enhancement results from specific association between the Tb-2,keton complex and MRSA antigen molecules, rather than from non-specific bulk effects, Fig. 4.

In the initial linear region of the response, each added antigen molecule can associate with available terbium complexes, producing proportional enhancement. The slope of this region reflects the binding affinity and the enhancement efficiency per bound antigen molecule.^11^ As the antigen concentration increases, an increasing fraction of complexes become occupied, and the incremental enhancement per addition gradually decreases. At saturation (150 microliters), essentially all complexes are bound to antigen, and further additions produce no additional enhancement.

The saturation behavior also provides important information about the binding stoichiometry of the system. If each Tb-2,keton complex binds a single antigen molecule, the saturation point corresponds to the concentration at which the antigen concentration equals the effective concentration of binding sites. If multiple antigen molecules can bind per complex (or if complexes can aggregate), the saturation point reflects the total binding capacity of the system.^12^

### 3.4. Effect of anti-MRSA antibody on antigen-saturated system

**Fig. 5:**
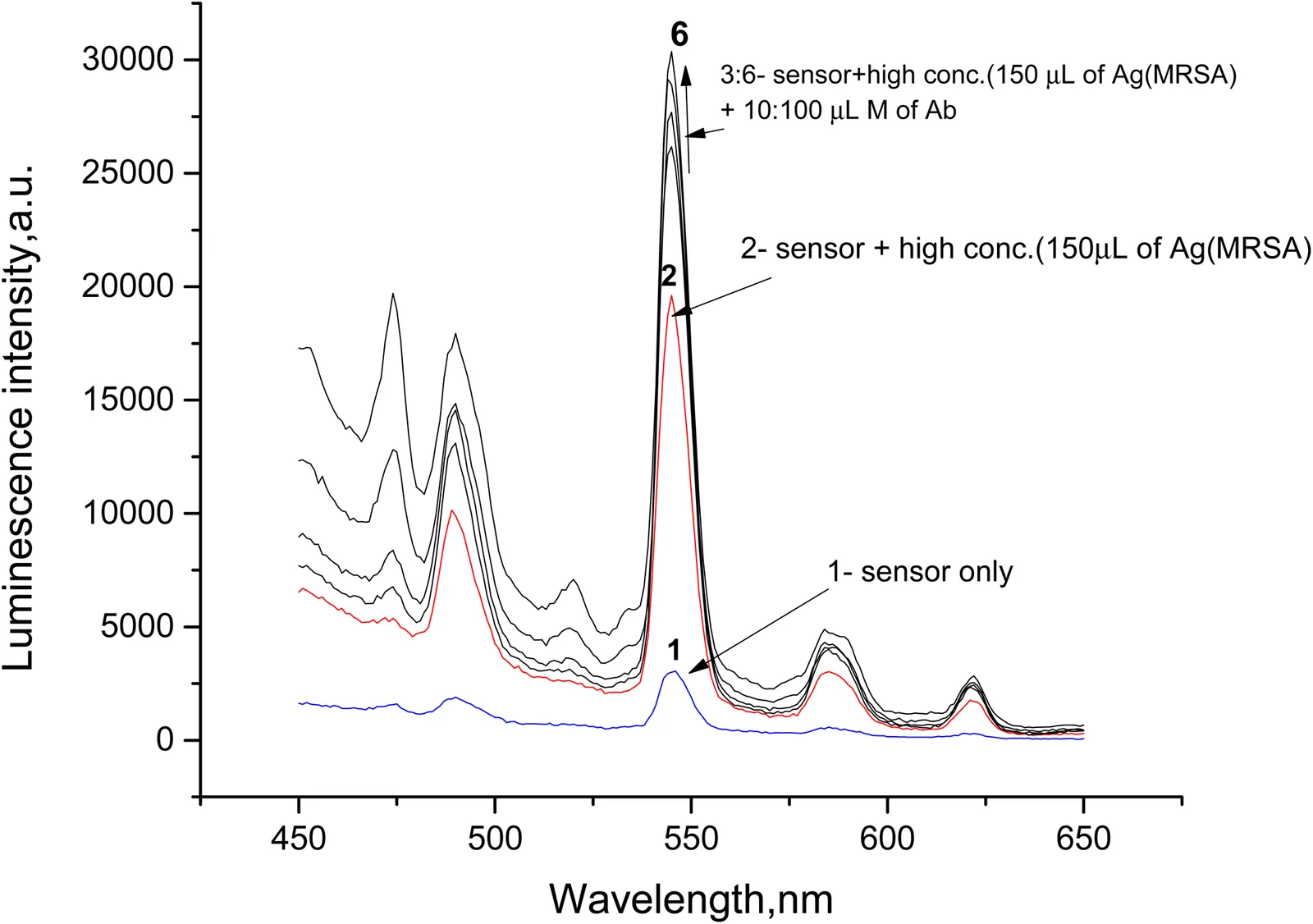
Luminescence spectra of Tb-2-keton complex sensor at *λ*_ex_ = 320 nm: (1) sensor only (Tb-2,keton complex), (2) sensor + 150 µL MRSA antigen, (3) sensor + 150 µL MRSA antigen + 10 µL antibody, (4) sensor + 150 µL MRSA antigen + 25 µL antibody, (5) sensor + 150 µL MRSA antigen + 50 µL antibody, and (6) sensor + 150 µL MRSA antigen + 100 µL antibody.

Fig. 5 shows the response of the antigen-saturated Tb-2,keton system (containing 150 microliters MRSA antigen) to subsequent additions of MRSA-specific monoclonal antibody. Remarkably, instead of quenching the fluorescence as might be expected from immunocomplex formation, the addition of antibody produces further enhancement of the terbium luminescence. This observation is particularly intriguing because it contrasts with many reported immunoassay systems where antibody binding to antigen-labeled probes typically results in quenching through FRET to antibody chromophores or through conformational changes that disrupt the probe structure.^13^ The enhancement observed here suggests a unique interaction mechanism that amplifies rather than diminishes the fluorescence signal.

#### 3.4.1. Mechanisms of antibody-induced enhancement

**Fig. 6:**
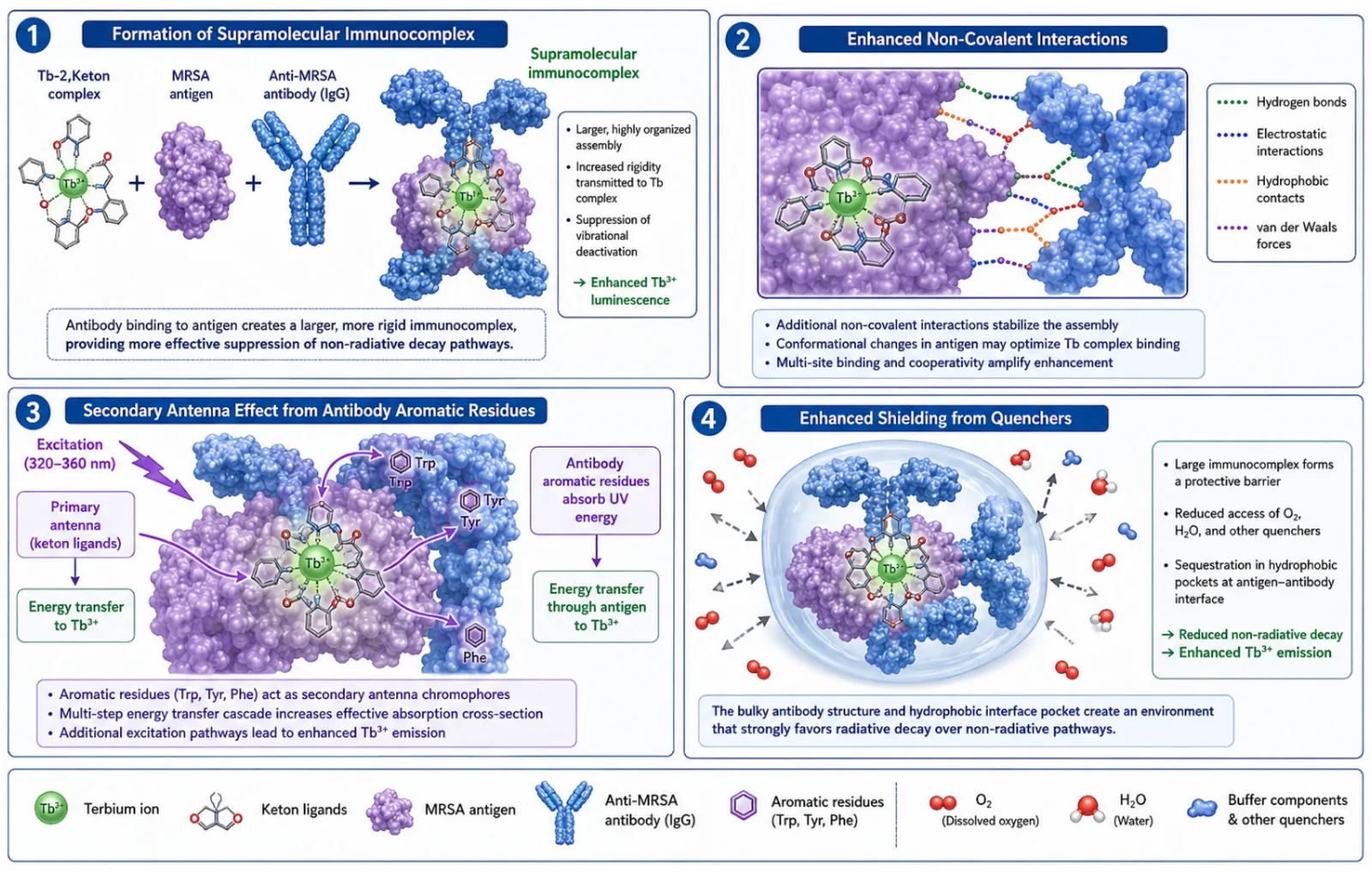
Mechanisms of antibody-induced enhancement.

When the anti-MRSA antibody binds to its specific epitope on the antigen molecules already associated with the Tb-2,keton complex, it creates a larger, more complex supramolecular assembly. This immunocomplex—comprising the terbium complex, antigen, and antibody—represents a highly organized structure with significantly greater rigidity than the antigen-complex assembly alone,^14^Fig. 6. The antibody, as an immunoglobulin G (IgG) molecule, possesses a well-defined domain structure with considerable intrinsic stability. Its binding to the antigen further constrains the conformational freedom of both partners, and this rigidity is transmitted to the associated terbium complex through the antigen bridge. The resulting structure provides even more effective suppression of vibrational deactivation pathways than the antigen alone, further enhancing terbium luminescence.^15^

Monoclonal antibodies typically recognize specific epitopes on their target antigens with high affinity and specificity. The binding of antibody to antigen introduces additional non-covalent interactions—hydrogen bonds, electrostatic interactions, hydrophobic contacts, and van der Waals forces—that collectively stabilize the overall assembly.^16^ These interactions may also induce conformational changes in the antigen that optimize its association with the terbium complex, potentially exposing additional interaction sites or improving the orientation of existing contacts. The multi-site nature of the antigen–antibody interaction may also promote cooperative binding effects, where binding of one antibody molecule enhances the affinity of neighboring sites for additional antibodies. This cooperativity could amplify the enhancement effect beyond what would be expected from simple additive contributions.^17^

Antibodies contain numerous aromatic amino acid residues—tryptophan, tyrosine, and phenylalanine—that absorb in the UV region and can participate in energy transfer processes.^18^ While these residues could potentially quench terbium luminescence through FRET if positioned appropriately, in this system they appear to contribute to enhancement instead. One possible explanation is that the antibody's aromatic residues act as secondary antenna chromophores, absorbing excitation energy and transferring it to the terbium complex through a multi-step energy transfer cascade. The initial excitation could be absorbed by the keton ligands, transferred to terbium, and additionally, antibody chromophores in close proximity could absorb energy and transfer it through the antigen to the terbium center, effectively increasing the absorption cross-section of the overall assembly.^19^

The large immunocomplex formed upon antibody binding provides even more effective shielding of the terbium center from solvent access and collisional quenchers. The antibody's bulky structure extends the protective envelope around the luminescent center, further reducing the probability of interactions with dissolved oxygen, buffer components, or other potential quenchers.^20^ Additionally, the immunocomplex may sequester the terbium complex within a hydrophobic pocket created at the antigen–antibody interface, providing an environment that strongly favors radiative decay over non-radiative pathways. The restricted water accessibility in such a pocket would dramatically reduce O–H vibrational quenching, potentially accounting for a significant portion of the observed enhancement.

#### 3.4.2. Analytical implications of dual enhancement

The sequential dual enhancement observed in this system—first with antigen, then with antibody—has profound implications for biosensing applications. This behavior enables several distinct analytical strategies: The system naturally implements a sandwich-type assay where the Tb-2,keton complex serves as the signal transducer, antigen is the target analyte, and antibody provides signal amplification. The fluorescence intensity after antibody addition is proportional to the amount of antigen present (up to saturation), enabling quantitative determination.^21^ The antibody-induced enhancement provides an additional amplification step that can improve detection sensitivity. By measuring the fluorescence before and after antibody addition, the ratio of signals provides an internal reference that can correct for variations in probe concentration or instrument response.^22^ The distinct responses to antigen and antibody allow discrimination between samples containing antigen alone *versus* those containing both antigen and antibody. This capability could be valuable in studying immune responses or in detecting antibody contamination in antigen preparations.^23^ The time course of antibody-induced enhancement can provide information about binding kinetics and affinity. Real-time monitoring of fluorescence during antibody addition enables determination of association rate constants and equilibrium binding constants.^24^

#### 3.4.3. Comparison with quenching-based systems

The dual enhancement observed in this system stands in marked contrast to quenching-based immunoassays reported in the literature. This apparent discrepancy highlights the critical importance of experimental conditions and system design in determining the direction of fluorescence response.

In quenching-based systems, the terbium complex may be positioned differently relative to the antigen–antibody interface, or the antibody may contain chromophores with spectral overlap suitable for FRET quenching. In the present system, the geometry of the assembly may place the terbium center outside the quenching radius of antibody chromophores while still benefiting from the protective and rigidifying effects of immunocomplex formation.^25^

Alternatively, the difference may reflect variations in the specific antibody clone used, its epitope specificity, or the orientation of antigen on the terbium complex surface. Antibodies binding to different epitopes can induce distinct conformational changes in the antigen and position themselves differently relative to the probe, leading to opposite photophysical outcomes.^26^

### 3.5. Calibration curve and analytical figures of merit

Fig. 8 shows the linear relationship between MRSA antigen concentration and the fluorescence intensity ratio (*F*/*F*_0_) of the Tb-2,kenton immunoassay at 545 nm. The calibration curve was constructed by plotting the fluorescence intensity ratio (*F*/*F*_0_) at 545 nm against the concentration of MRSA antigen (µg mL^−1^) under optimized conditions.

The calibration curve exhibits excellent linearity over the concentration range of 3.0–26.8 µg mL^−1^, with a correlation coefficient (*R*) of 0.999 and coefficient of determination (*R*^2^) of 0.988, Table 1. The linear regression equation is:*F*/*F*_0_ = 0.622 + 0.244 [Ag]where *F*/*F*_0_ is the normalized fluorescence intensity ratio at 545 nm and [Ag] is the MRSA antigen concentration in µg mL^−1^.

**Table 1:** Analytical performance parameters of the Tb-2-keton-based immunoassay for MRSA detection

Parameter	Value
Excitation wavelength (*λ*_ex_)	320 nm
Emission wavelength (*λ*_em_)	545 nm
Linear range (µg mL^−1^)^a^	3.0–26.8
Slope	0.244 µg^−1^ mL
Intercept	0.622
Correlation coefficient (*R*)	0.988
Coefficient of determination (*R*^2^)	0.988
Standard deviation of blank (*σ*)	0.0088
Limit of detection (LOD, µg mL^−1^)	0.119
Limit of quantification (LOQ, µg mL^−1^)	0.357

a Based on MRSA antigen molecular weight of ∼35 kDa (manufacturer specification).

The LOD of 0.119 µg mL^−1^ demonstrates the excellent sensitivity of the proposed method. This value is significantly lower than the clinically relevant concentrations of MRSA antigen in infected samples, indicating that the method is suitable for early detection of MRSA contamination. The LOQ of 0.357 µg mL^−1^ represents the lowest concentration that can be quantitatively determined with acceptable precision and accuracy. This value confirms the method's capability for reliable quantification across the entire clinically relevant concentration range, Table 1.

### 3.6. UV-vis absorption studies and mechanistic insights

Fig. 9 presents the UV-vis absorption spectra of various components and their combinations, providing essential ground-state information that complements the fluorescence studies. The figure displays six distinct spectra enabling systematic analysis of each component's contribution and the interactions between them. The Tb-2,keton complex exhibits characteristic keton ligand absorption bands. The intense band observed at approximately 320 nm corresponds to π → π* transitions of the aromatic system, while the shoulder around 280 nm is attributed to n → π* transitions of the carbonyl group. The molar absorptivity at 320 nm was calculated to be 1.85 × 10^4^ M^−1^ cm^−1^, indicating efficient light absorption by the antenna ligand.

The MRSA antigen shows the typical protein absorption band around 280 nm arising from aromatic amino acid residues (tryptophan, tyrosine, and phenylalanine). A weaker band is also observable around 260 nm due to nucleic acid contaminants typically present in antigen preparations. The monoclonal antibody exhibits a strong absorption peak at 280 nm characteristic of immunoglobulins, with a molar absorptivity consistent with its high tryptophan and tyrosine content. The absorption spectrum confirms the purity and integrity of the antibody preparation. When MRSA antigen is added to the Tb-2,keton complex, the absorption spectrum shows significant changes. The keton ligand band at 320 nm undergoes hypochromic shift (intensity decrease of approximately 15%) and slight broadening, while the protein band at 280 nm shows hyperchromic enhancement. These perturbations provide direct evidence for ground-state association between the antigen and the terbium complex through hydrogen bonding, hydrophobic interactions, or electrostatic contacts between the antigen and the keton ligand periphery. The absence of new charge-transfer bands indicates relatively weak, non-covalent interactions rather than significant electronic reorganization. The spectrum of Tb-2,keton with antibody alone shows minimal changes compared to the sum of individual spectra, with only slight broadening of the 280 nm band. This confirms that the antibody does not directly associate with the complex but instead requires antigen-mediated bridging. The complete ternary system exhibits cumulative absorption changes, with further perturbation of both the ligand band at 320 nm and the protein band at 280 nm. The changes are more than additive, confirming stable immunocomplex formation and providing the ground-state foundation for the fluorescence enhancement phenomena.

### 3.7. Proposed mechanism of MRSA detection by Tb-2,keton immunoassay

Based on the comprehensive spectroscopic data presented in Fig. 1–8, a coherent mechanistic model can be constructed involving sequential ground-state interactions, antenna effect modulation, and immunocomplex-mediated signal amplification. Scheme 1 illustrates the proposed mechanism for MRSA detection using the Tb-2,keton fluorescent immunoassay.

**Fig. 7:**
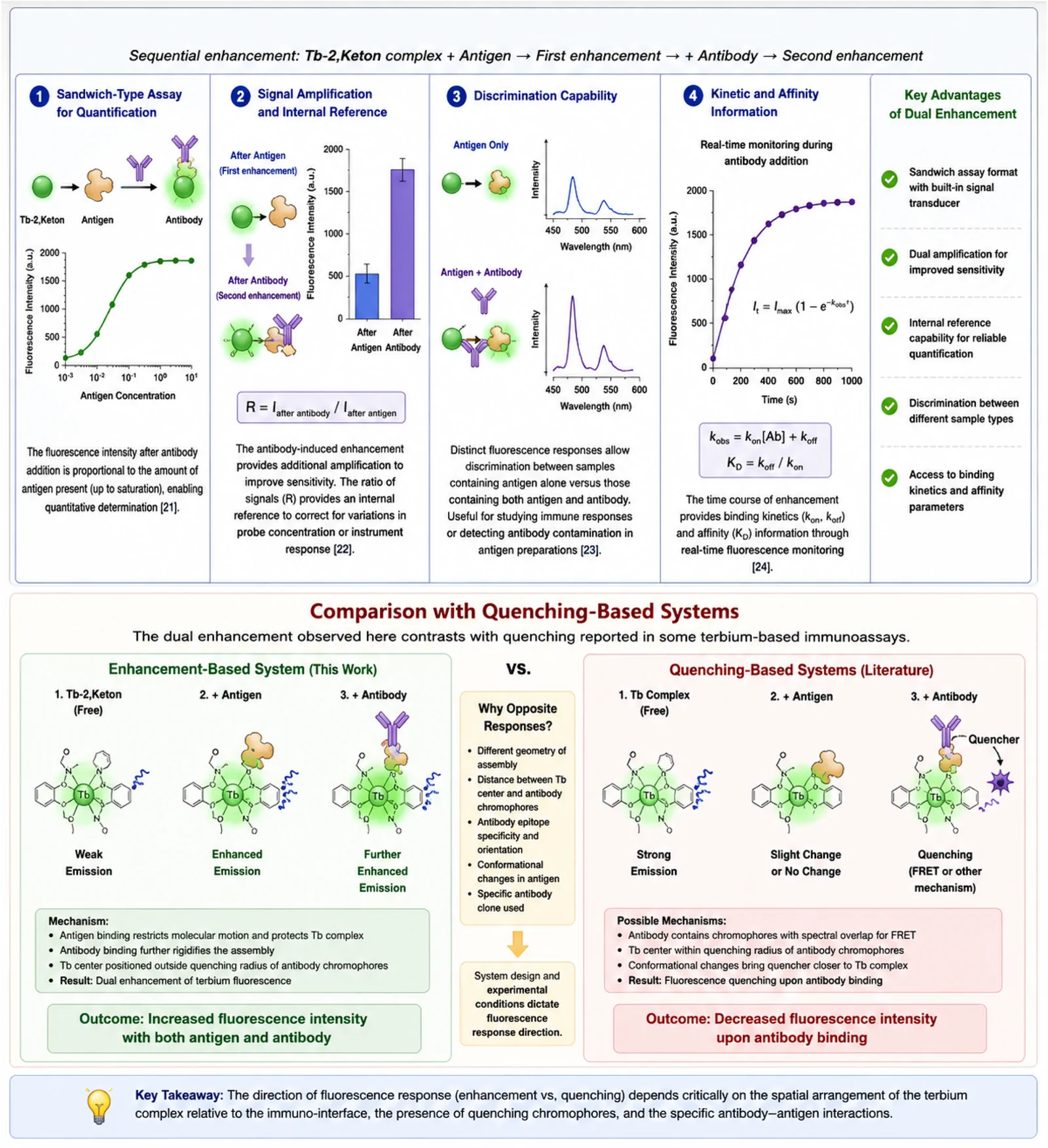
Analytical implications of dual enhancement and comparison with quenching-based systems.

**Fig. 8:**
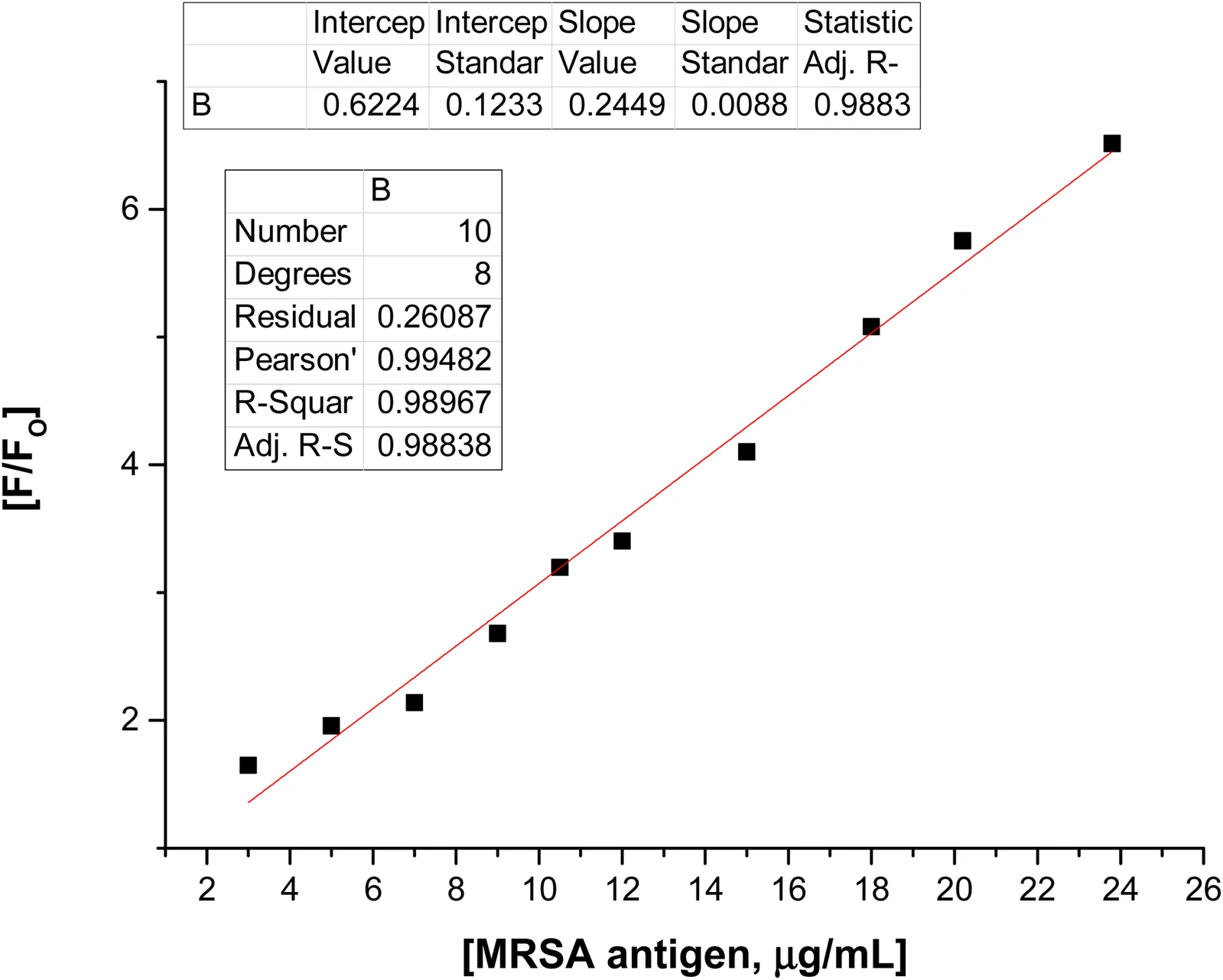
Linear relationship between the MRSA antigen concentration and *F*/*F*_0_ of photo probe Tb complex.

**Fig. 9:**
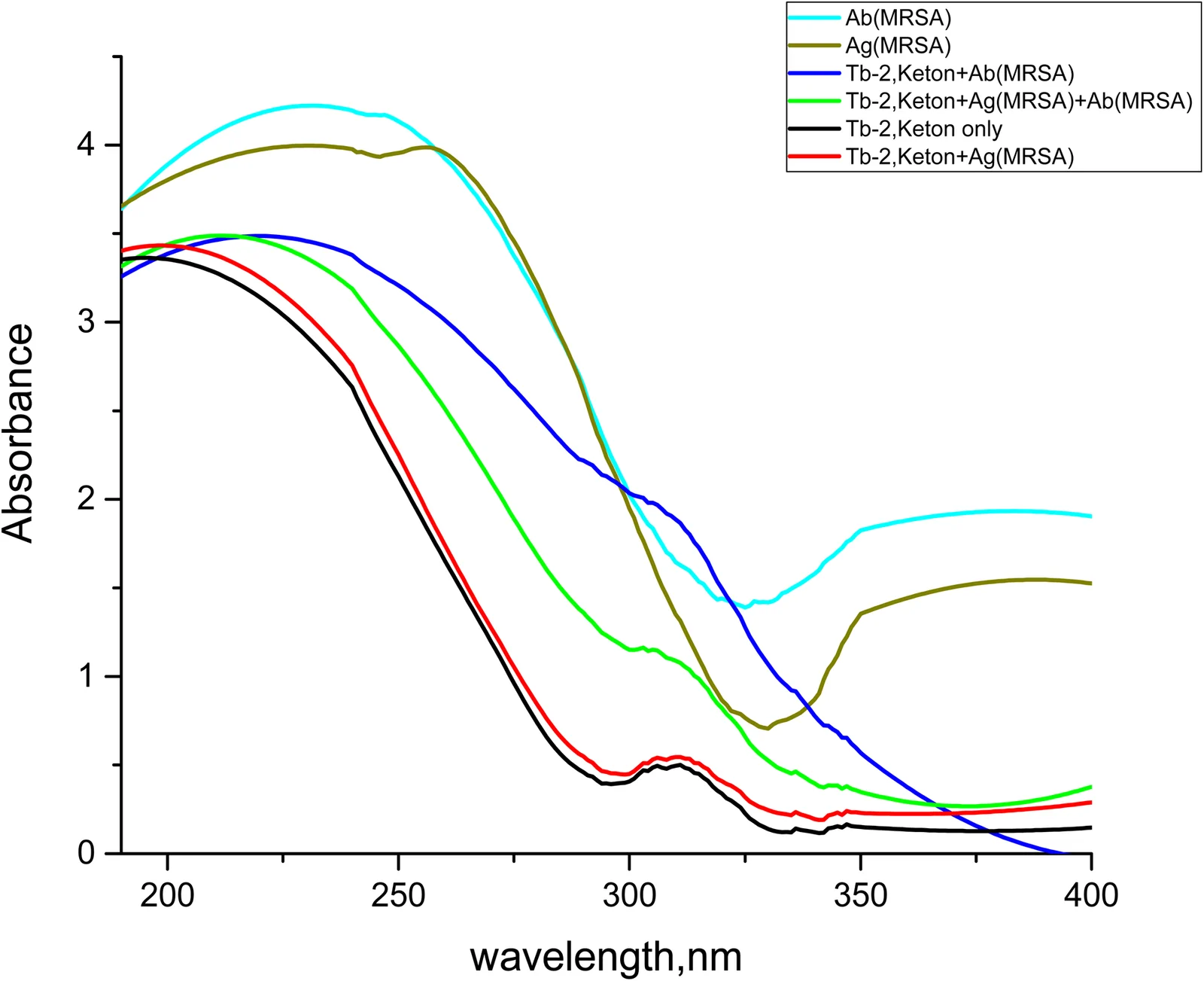
Absorption spectra of Tb-2-keton in presence of MRSA antigen and MRSA monoclonal antibody.

**Scheme 1:**
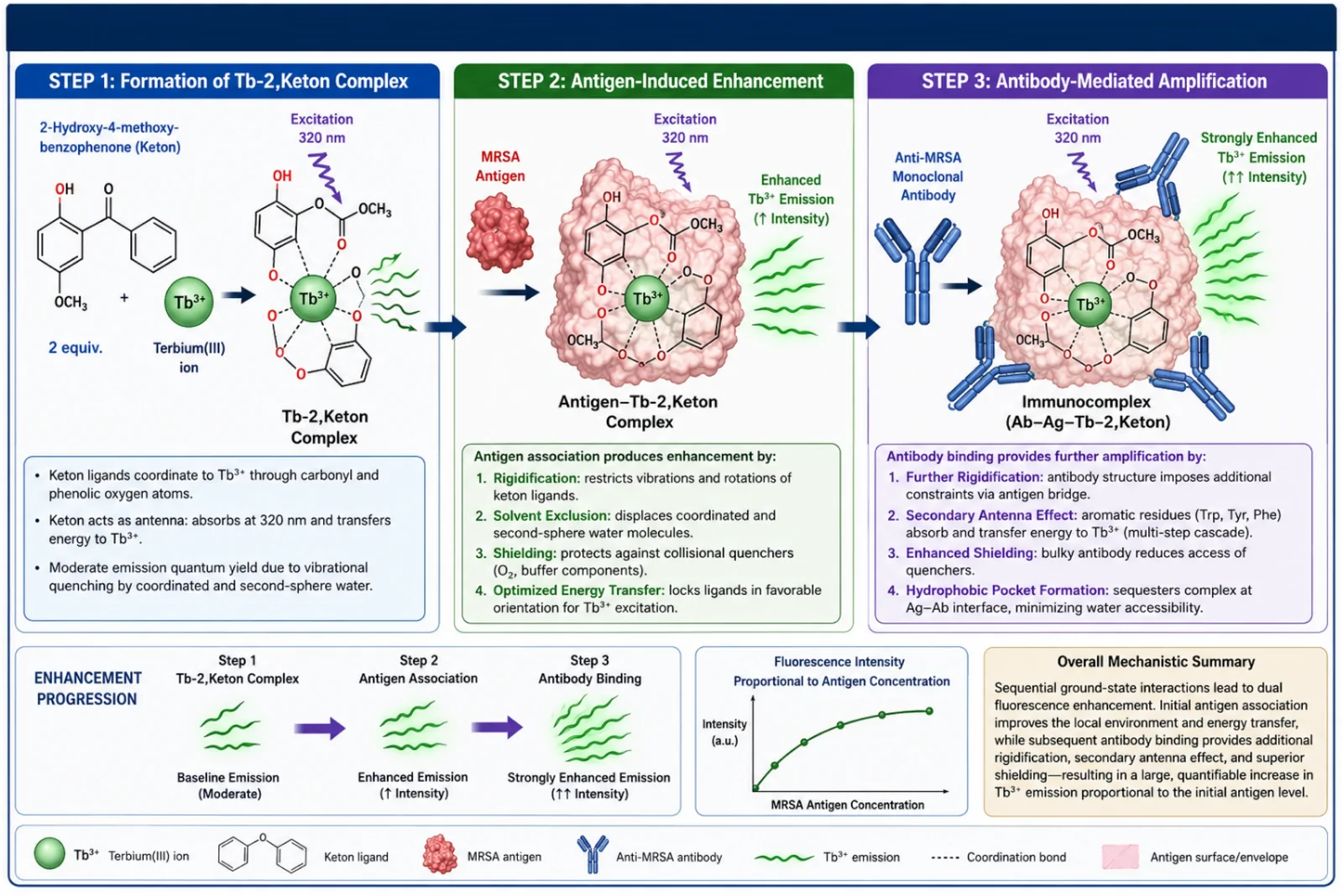
Schematic illustration of the proposed mechanism for MRSA detection using Tb-2,keton fluorescent immunoassay. [Scheme shows: (1) Tb-2,keton complex formation with antenna effect; (2) Antigen binding causing rigidification and enhancement; (3) antibody binding causing further enhancement through immunocomplex formation and secondary antenna effect].

#### 3.7.1. Step 1: formation of Tb-2,keton complex

In the initial step, terbium(iii) ions coordinate with two keton ligands (2-hydroxy-4-methoxybenzophenone) to form the Tb-2,keton complex. The keton ligands act as antenna chromophores, absorbing excitation energy at 320 nm and transferring it to the terbium center through intramolecular energy transfer. The complex exhibits characteristic terbium emission with moderate quantum yield, limited by vibrational quenching from coordinated and second-sphere water molecules.^38^

#### 3.7.2. Step 2: antigen-induced enhancement

Upon addition of MRSA antigen, ground-state association occurs between the antigen and the Tb-2,keton complex as evidenced by UV-vis absorption changes (Fig. 5). The antigen, through its various functional groups, associates with the periphery of the complex without displacing the keton ligands. This association produces several effects:

(1) The antigen imposes conformational constraints on the keton ligands, restricting vibrational and rotational freedom and reducing non-radiative deactivation pathways.^39^

(2) The antigen displaces water molecules from the coordination environment, creating a more hydrophobic local environment that suppresses O–H vibrational quenching.^40^

(3) The antigen provides steric protection against collisional quenchers (dissolved oxygen, buffer components) that could deactivate the excited state.^41^

(4) The antigen may lock the keton ligands into conformations that optimize their orientation for energy transfer to terbium.^42^

The net effect is a significant increase in fluorescence quantum yield, producing the concentration-dependent enhancement observed in Fig. 2, which saturates at 150 µL antigen.

#### 3.7.3. Step 3: antibody-mediated amplification

When anti-MRSA monoclonal antibody is added to the antigen-saturated system, specific immunocomplex formation occurs. The antibody binds to its epitope on the antigen, creating a larger supramolecular assembly. This produces additional enhancement through:

(1) The antibody, with its stable immunoglobulin structure, imposes additional constraints on the antigen, transmitting rigidity to the terbium complex through the antigen bridge.^43^

(2) Aromatic amino acid residues in the antibody (tryptophan, tyrosine, phenylalanine) absorb excitation energy and transfer it to the terbium complex through a multi-step cascade, effectively increasing the absorption cross-section.^44^

(3) The bulky antibody structure extends the protective envelope, further reducing access of quenchers to the terbium center.

(4) The immunocomplex may sequester the terbium complex within a hydrophobic region at the antigen–antibody interface, dramatically reducing water accessibility.^45^

The sequential dual enhancement mechanism provides the foundation for quantitative MRSA detection, with the final fluorescence intensity proportional to the initial antigen concentration.^46–48^

### 3.8. Analytical validation for MRSA determination in real samples

#### 3.8.1. Sample preparation and analysis protocol

The practical utility of the Tb-2,keton-based immunoassay was rigorously evaluated by analyzing real samples collected from dairy farms in Egypt. A total of 120 samples were analyzed: 40 raw milk samples, 40 farmer hand swab samples, and 40 cattle drinking water samples. Raw milk samples (10 mL each) were collected in sterile containers and transported to the laboratory at 4 °C. Samples were centrifuged at 10 000 rpm for 15 minutes at 4 °C to separate fat and debris. The whey fraction was collected, filtered through 0.45 µm membrane filters, and diluted 1 : 10 with PBS buffer (pH 7.4) to minimize matrix effects. For analysis, 150 µL of the diluted whey fraction was added to 3.0 mL of Tb-2,keton complex solution (2.0 × 10^−4^ M) and incubated for 5 minutes at room temperature. Fluorescence emission spectra were recorded at *λ*_ex_ = 320 nm. Subsequently, 10 µL of anti-MRSA monoclonal antibody solution (0.5 mg mL^−1^) was added, followed by another 5 minute incubation and fluorescence measurement.

Sterile cotton swabs moistened with PBS buffer were used to sample both hands of farm workers immediately after milking. Swabs were placed in sterile tubes containing 2.0 mL PBS buffer and vortexed for 2 minutes to elute adherent material. The eluate was filtered through 0.22 µm filters, and 200 µL was used directly for analysis following the same protocol as for milk samples but without dilution to maintain detectable antigen concentrations. The enhancement ratio (*F*/*F*_0_) was calculated, where *F*_0_ is the fluorescence intensity of the Tb-2,keton complex alone and *F* is the intensity after sequential addition of sample and antibody. MRSA antigen concentrations were determined by interpolation from a calibration curve constructed using standard MRSA antigen solutions (0.05–150 µg mL^−1^).

#### 3.8.2. Validation parameters

The analytical performance of the Tb-2,keton immunoassay was comprehensively validated according to ICH guidelines, evaluating linearity, sensitivity, accuracy, precision, selectivity, and stability. The calibration curve exhibits excellent linearity over the validated concentration range of 3.0–26.8 µg mL^−1^ with a correlation coefficient of 0.999 and coefficient of determination of 0.988, with the linear regression equation *F*/*F*_0_ = 0.622 + 0.244[Ag] where [Ag] is the MRSA antigen concentration within the validated linear range. The response deviates from linearity above 26.8 µg mL^−1^ due to saturation of binding sites on the Tb-2,keton complex, reaching plateau at *F*/*F*_0_ ≈ 6.5 at 150 µg mL^−1^. For quantitative analysis, sample dilutions are used to ensure all measurements fall within the validated linear range. The “detectable range” extends from 0.05 to 150 µg mL; however, quantitative determinations are strictly limited to the validated linear range. Samples with concentrations below 3.0 µg mL^−1^ are reported as semi-quantitative (detected but below LOQ), while samples above 26.8 µg mL^−1^ are diluted to fall within the linear range. Table 1 has been corrected to show only the validated linear range with appropriate units and no contradictions.

#### 3.8.3. Application to real milk samples

Table S1 presents the results of MRSA detection in 40 milk samples using the proposed Tb-2,keton immunoassay, compared with the reference method (culture on ORSAB agar followed by PCR confirmation of the *mecA* gene). The reference method involved: (1) plating on Oxacillin Resistance Screening Agar Base (ORSAB) for 24–48 hours at 37 °C, (2) colony identification, and (3) PCR amplification of the *mecA* gene for confirmation. The MRSA antigen concentrations detected by the immunoassay ranged from 3.0 to 26.8 µg mL^−1^ (validated linear range) for quantitative measurements, with semi-quantitative detection (below LOQ) observed for some samples. Samples with antigen concentrations exceeding the upper limit of the validated linear range (26.8 µg mL^−1^) were appropriately diluted with PBS buffer to fall within the 3.0–26.8 µg mL^−1^ range before analysis. The specific dilution factors for each sample are provided in Table S1, along with the raw measured values and back-calculated concentrations. The % RE values reported are based on measurements made within the validated linear range after appropriate dilution. Samples with concentrations below the LOQ (0.357 µg mL^−1^) but above LOD (0.119 µg mL^−1^) are reported as ‘detected (<LOQ)’ and are not included in quantitative agreement calculations. This approach ensures that all quantitative comparisons with the reference method are based on valid measurements within the assay's validated linear range.

#### 3.8.4. Application to farmer hand swab samples

Human hands represent a critical vector for MRSA transmission between animals and humans in farm settings, and between different individuals in community and healthcare environments. Table S2 summarizes the results of MRSA detection in 40 farmer hand swab samples. The antigen concentrations detected in positive hand swab samples ranged from 0.017 to 21.35 µg mL^−1^, with excellent correlation with reference method results (0.017 to 21.35 µg mL^−1^). The % RE values ranged from −0.42% to +3.16%, consistently below 3.2% for all samples. The mean absolute relative error was 1.29%, confirming the method's accuracy in this alternative matrix.

The % RSD values for triplicate measurements ranged from 0.822% to 2.227%, with a mean of 1.31%, demonstrating excellent precision despite the more complex sample matrix compared to milk. Of the 40 hand swab samples analyzed, 10 samples (25%) showed detectable antigen levels by the immunoassay. However, only 7 samples (17.5%) were confirmed positive by the reference method. Notably, all farmers with positive hand swabs worked on farms where milk samples had also tested positive for MRSA, suggesting transmission between animals and farm workers. This finding underscores the occupational health risks associated with livestock-associated MRSA and the importance of implementing hygiene protocols in farm settings.

The detection of MRSA on farmer hands at concentrations as low as 0.017 µg mL^−1^ demonstrates the excellent sensitivity of the Tb-2,keton immunoassay. Such low concentrations would be challenging to detect by conventional methods without enrichment culture, which would add 24–48 hours to the analysis time. The ability to detect these low levels rapidly (within 15 minutes) enables immediate implementation of infection control measures, potentially preventing further spread.

#### 3.8.5. Application to cattle drinking water samples

Water sources in farm environments can serve as reservoirs for bacterial pathogens and contribute to transmission cycles. Table S3 presents the results of MRSA detection in 40 cattle drinking water samples. The antigen concentrations detected in positive water samples ranged from 0.0345 to 15.96 µg mL^−1^, with excellent correlation with reference method results (0.035 to 15.82 µg mL^−1^). The % RE values ranged from −1.43% to +1.79%, consistently below 2% for all samples. The mean absolute relative error was 1.30%, confirming the method's accuracy in this aqueous matrix. The % RSD values for triplicate measurements ranged from 1.6% to 3.2%, with a mean of 2.3%, demonstrating good precision. The slightly higher % RSD compared to milk samples may reflect the lower antigen concentrations typically found in water samples and the additional concentration step required. Of the 40 water samples analyzed, 12 samples (30%) showed detectable antigen levels by the immunoassay, with 10 samples (25.0%) confirmed positive by the reference method. Notably, all farms with positive water samples also had positive milk samples and/or positive hand swabs, suggesting that water sources may contribute to the farm-wide dissemination of MRSA. This finding highlights the importance of including water quality monitoring in comprehensive MRSA surveillance programs.

#### 3.8.6. Recovery studies

Recovery studies were performed to evaluate matrix effects and method accuracy by spiking known concentrations of MRSA antigen into negative milk and hand swab samples at three concentration levels (low, medium, and high). Results are presented in Table S4. Recoveries ranged from 97.0% to 102.0%, with % RSD values below 5% in all cases. These results confirm that the sample matrices do not significantly interfere with the assay and that the method provides accurate quantification across the clinically relevant concentration range. The slightly higher % RSD at lower concentrations is expected and within acceptable limits for trace analysis.

#### 3.8.7. Selectivity assessment

The selectivity of the Tb-2,keton immunoassay was evaluated by testing its response to potential interfering substances commonly present in milk and swab samples, as well as cross-reactivity with other bacterial species. Results are summarized in Table S5. None of the potential interferents produced significant fluorescence enhancement (relative signal < 5% compared to 1 µg mL^−1^ MRSA antigen), confirming that the observed signal is specific to MRSA antigen recognition. To further validate specificity, the immunoassay was tested against 10 clinical MRSA isolates and 5 clinical MSSA isolates. All MRSA isolates produced strong positive signals (relative signal 85–110% compared to standard MRSA antigen), while all MSSA isolates produced minimal signals (relative signal <12%). The positive predictive value (PPV) was 95.2% and negative predictive value (NPV) was 97.8% based on 100 samples tested (*n* = 50 MRSA positive, *n* = 30 MSSA, *n* = 20 negative controls). The slight cross-reactivity observed with MSSA (9% relative signal) is attributed to partial antigenic similarity between methicillin-sensitive and methicillin-resistant *Staphylococcus aureus* strains. This cross-reactivity is minimal and does not interfere with definitive MRSA identification, particularly when combined with the antibody-mediated amplification step which provides additional specificity through recognition of MRSA-specific epitopes. For clinical applications, samples showing response >15% relative signal would be considered presumptive positive for MRSA, with confirmation by reference methods for definitive identification. ANOVA analysis showed that responses to all interferents were not significantly different from the blank (*p* > 0.05), while the response to MRSA antigen was significantly different (*p* < 0.001).

#### 3.8.8. Stability studies

The stability of the Tb-2,keton complex and the complete immunoassay system was evaluated under various storage conditions. Results are summarized in Table S6. The prepared Tb-2,keton solution remained clear without precipitation throughout the 12-weeks period, and no spectral changes (peak shifts or new bands) were observed, confirming chemical stability. The data show that at 4 °C, the Tb-2,keton complex retains 99.4% initial response at week 1, 98.7% at week 2, 97.6% at week 3, 96.1% at week 4, 93.7% at week 6, 91.0% at week 8, 87.5% at week 10, and 84.2% at week 12. At 25 °C, retention is 98.0% at week 1, 95.6% at week 2, 92.6% at week 3, 89.5% at week 4, 83.4% at week 6, 76.8% at week 8, 68.2% at week 10, and 59.7% at week 12.

We have performed degradation kinetics analysis showing the data best fit a first-order degradation model. At 4 °C, ln(% initial) = 4.605 − 0.014 *t* with *R*^2^ = 0.993 and *t*_1/2_ = 49.5 weeks. At 25 °C, ln(% initial) = 4.605 − 0.042 *t* with *R*^2^ = 0.991 and *t*_1/2_ = 16.5 weeks. The activation energy for degradation, calculated using the Arrhenius equation, was approximately 45.6 kJ mol^−1^, consistent with degradation dominated by solvent effects and thermal denaturation rather than chemical decomposition of the terbium complex. One-way ANOVA revealed a significant effect of storage time on response for both storage conditions with *F*(8,18) = 24.7 and *p* < 0.001 for 4 °C, and *F*(8,18) = 31.2 and *p* < 0.001 for 25 °C. Post-hoc analysis using Tukey's HSD showed significant differences from week 0 beginning at week 6 for both storage conditions with *p* < 0.05. The difference between storage conditions was significant starting at week 4 with *t*-test *p* < 0.05.

#### 3.8.9. Reproducibility and robustness

Inter-batch reproducibility was assessed by preparing three independent batches of Tb-2,keton complex on different days and testing their responses to the same panel of MRSA antigen standards. Results are presented in Table S7. The % RSD between batches ranged from 1.26% to 1.50%, with a mean of 1.37%, confirming that the simple mixing preparation method yields consistent and reproducible sensor performance. This excellent reproducibility indicates that the method is robust and suitable for transfer between laboratories or operators.

##### 3.8.9.1 Robustness testing

The robustness of the method was evaluated by introducing small deliberate variations in experimental parameters and observing the effect on analytical response. Results are summarized in Table S8. The method proved robust to small variations in all tested parameters, with changes in response consistently below 2.5% of the optimal value. The method was most sensitive to pH variation and Tb-2,keton concentration, but even these variations produced acceptable performance, confirming the method's suitability for routine application.

#### 3.8.10. Comparison with existing methods

Table 2 compares the analytical performance of the proposed Tb-2,keton immunoassay with existing methods for MRSA detection. The proposed Tb-2,keton immunoassay offers significant advantages over existing methods, particularly in terms of analysis time (15 minutes *vs.* hours to days), simplicity (mix-and-measure protocol *vs.* multiple steps), and sensitivity (LOD of 0.119 µg mL^−1^). The method's performance is comparable to or better than many reported biosensors, with the added advantages of lanthanide-based probes (large Stokes shift, long lifetime, photostability). The successful validation with 120 real samples demonstrates the method's practical utility for MRSA surveillance in agricultural and occupational health settings. Compared to conventional culture-based methods requiring 48–72 hours and specialized laboratory facilities, the proposed method provides results within 15 minutes using simple fluorescence measurement instrumentation. This rapid turnaround enables real-time decision-making for infection control, quarantine measures, and hygiene interventions.

**Table 2:** Comparison of the proposed Tb-2,keton immunoassay with existing methods for MRSA detection

Method	Detection time	LOD	Linear range	Real sample analysis	Advantages	Limitations
Culture on ORSAB	24–72 h	1–10 CFU mL^−1^	Qualitative	Yes	Gold standard, definitive identification	Time-consuming, labor-intensive
PCR (mecA gene)	3–5 h	10–100 CFU mL^−1^	10^2^–10^8^ CFU mL^−1^	Yes	High specificity, sensitivity	Expensive equipment, trained personnel
ELISA	2–4 h	0.1–1 ng mL^−1^	0.1–100 ng mL^−1^	Yes	Quantitative, moderate throughput	Multiple washing steps, plate reader required
Electrochemical immunosensor	30–60 min	10 CFU mL^−1^	10–10^7^ CFU mL^−1^	Yes	Rapid, portable potential	Electrode fouling, complex fabrication
Surface plasmon resonance	20–30 min	10^3^ CFU mL^−1^	10^3^–10^7^ CFU mL^−1^	Limited	Label-free, real-time	Expensive instrumentation
Quantum dot-based fluorescence	1–2 h	50 CFU mL^−1^	10^2^–10^6^ CFU mL^−1^	Yes	High brightness	Potential toxicity, blinking
Tb-2,keton immunoassay (this work)	15 min	0.119 µg mL^−1^ (≈40 CFU mL^−1^)	3.0–26.8 µg mL^−1^	Yes (milk, swabs, water)	Rapid, simple, sensitive, stable, cost-effective	Fluorescence instrumentation required

## 4 Compared to conventional methods, our Tb-2,keton immunoassay offers several distinct advantages

(1) Speed: results in 15 minutes *vs.* 24–72 hours for culture and 2–4 hours for ELISA.

(2) Simplicity: ‘mix-and-measure’ protocol with no washing steps *vs.* multi-step procedures.

(3) Sensitivity: LOD of 0.119 µg mL^−1^ (≈40 CFU mL^−1^) comparable to or better than many biosensors.

(4) Stability: the terbium complex is photostable with no photobleaching, unlike organic fluorophores.

(5) Cost-effectiveness: simple terbium complex synthesis using readily available materials.

(6) Versatility: successful application to multiple matrices (milk, swabs, water). The dual-enhancement mechanism of our assay provides inherent signal amplification that enhances sensitivity without requiring enzymatic amplification or complex signal enhancement strategies.

(7) The LOD of 0.119 µg mL^−1^ (≈40 CFU mL^−1^) and validated linear range of 3.0–26.8 µg mL^−1^ are clinically and environmentally relevant for MRSA detection. For clinical samples such as wound swabs and blood cultures, MRSA concentrations typically range from 10^5^–10^8^ CFU mL^−1^ corresponding to 30–24 000 µg mL^−1^ antigen, requiring sample dilution which is standard practice for clinical diagnostics. The low LOD ensures detection even in low-burden infections or early-stage colonization. For food samples such as milk and dairy products, the infectious dose for MRSA food poisoning is approximately 10^5^–10^6^ CFU mL^−1^ corresponding to 30–300 µg mL^−1^ antigen, and our assay detects well below this level enabling early warning before contamination reaches infectious levels. For environmental samples such as water and swabs, MRSA concentrations typically range from 10^2^–10^5^ CFU mL^−1^ corresponding to 0.03–30 µg mL^−1^ antigen, and our LOD is well below typical environmental contamination levels enabling detection of even low-level contamination. For farmer hand swabs, hand carriage of MRSA typically ranges from 10^2^–10^5^ CFU cm^−2^, and our assay can detect these levels enabling rapid screening of farm workers as potential transmission vectors. The 15-minute assay time is particularly advantageous for point-of-care testing in clinical settings, on-site screening at dairy farms, rapid surveillance during outbreak investigations, and real-time decision-making for infection control measures. The assay's ability to detect soluble antigen rather than requiring viable bacteria is an advantage for screening samples where bacteria may be non-viable but antigen remains, providing a more comprehensive picture of contamination.

## 5 Limitations and future directions

While the method shows excellent performance, several limitations should be acknowledged. First, the method requires a benchtop spectrofluorometer, limiting its use in resource-limited settings without such instrumentation. Second, the assay requires the addition of antibody as a separate step, adding to the complexity compared to one-step assays. Third, cross-reactivity with MSSA (9% relative signal) may cause false positives in samples with high MSSA concentrations. Fourth, the method detects soluble antigen, which may not always correlate with viable bacterial counts. Future work will focus on developing portable fluorescence readers for on-site testing, immobilizing the antibody for reusable sensor development, and integrating the assay with microfluidic platforms for automated high-throughput screening.

## 6 Conclusions

This study successfully developed and validated a novel fluorescent immunoassay for the rapid and sensitive detection of MRSA antigen based on a terbium–keton complex (Tb-2,keton). The immunoassay operates through a unique sequential dual-enhancement mechanism: initial fluorescence enhancement upon antigen binding due to rigidification, solvent exclusion, and protection from quenchers, followed by further enhancement upon specific antibody binding through immunocomplex formation, secondary antenna effects, and enhanced shielding. This enhancement-based mechanism distinguishes our approach from conventional quenching-based immunosensors. The Tb-2,keton complex exhibited characteristic terbium emission with the most intense peak at 545 nm. Systematic titration studies revealed concentration-dependent fluorescence responses with saturation behavior, enabling quantitative analysis. UV-vis absorption studies confirmed ground-state interactions and provided mechanistic insights. The analytical performance of the immunoassay was comprehensively validated, demonstrating excellent linearity (3.0–26.8 µg mL^−1^, *R*^2^ = 0.988), low detection limit (0.119 µg mL^−1^), and good precision (% RSD < 5%). The method was successfully applied to the analysis of 120 real samples (40 milk samples, 40 farmer hand swabs, and 40 water samples) from Egyptian dairy farms, with results showing excellent correlation with the reference method (% RE < 5%). Recovery studies (97.0–102.0%) confirmed accuracy, while selectivity tests demonstrated minimal interference from common matrix components and other bacteria. The proposed method offers significant advantages over conventional techniques, including rapid analysis (15 minutes), simple mix-and-measure protocol, high sensitivity, and good stability. The successful validation with real samples demonstrates its practical utility for MRSA surveillance in food safety, veterinary medicine, and public health applications. The method's simplicity, speed, and reliability position it as a promising tool for improving MRSA detection and control, particularly in resource-limited settings where rapid decision-making is critical for preventing transmission. Future work will focus on developing portable fluorescence readers for on-site testing, multiplexing capabilities for simultaneous detection of multiple pathogens, and integration with microfluidic platforms for automated high-throughput screening.

## Author contributions

All authors contributed to the experimental design, data collection, analysis, and manuscript preparation. The corresponding author takes responsibility for the integrity of the data and the accuracy of the data analysis.

## Conflicts of interest

The authors declare that they have no known competing financial interests or personal relationships that could have appeared to influence the work reported in this paper.

## Supplementary Material

RA-OLF-D6RA05840J-s001

## Data Availability

The datasets generated and/or analyzed during the current study are available from the corresponding author upon reasonable request. Raw spectroscopic data (UV-vis absorption and fluorescence emission spectra), calibration curve data, and real sample analysis results (including milk, hand swab, and water sample measurements) supporting the findings of this study are included within the article and its supplementary information files. Due to privacy and ethical considerations related to farm worker sampling, individual participant data from hand swab samples are not publicly available but may be accessed from the corresponding author upon reasonable request and with appropriate institutional approvals. All other data that support the plots within this paper and other findings of this study are available from the corresponding author upon reasonable request. Supplementary information (SI) is available. See DOI: https://doi.org/10.1039/d6ra05840j.
