# Supplementary material for: A highly sensitive terbium–keton complex-based fluorescent immunoassay for detection of methicillin-resistant Staphylococcus aureus (MRSA) antigen in milk, farmer hand swabs, and cattle drinking water
Source: RSC Adv. 2026 Sep 14;16(44):54820–38. doi: 10.1039/d6ra05840j (PMC13573165; doi:10.1039/d6ra05840j)
Supplement: Supplementary file 1 [file RA-016-D6RA05840J-s001.pdf]

This journal is © The Royal Society of Chemistry 2026

Table S1: Determination of MRSA in real milk samples using the proposed Tb-2,Keton immunoassay compared with reference method.

Sample	Reference Method (µg/mL)	Dilution Factor	Raw Measured (µg/mL)	Back-Calculated (µg/mL)	%RE	%RSD	Confirmed
1	120.1	5	24.02 ± 0.14	120.10 ± 0.72	0.00	0.60	Yes
2	0.24	1	0.23 ± 0.01	0.23 ± 0.01	-4.76	4.10	Yes*
3	38.8	2	19.40 ± 0.37	38.80 ± 0.74	0.00	1.90	Yes
4	0.31	1	0.32 ± 0.01	0.32 ± 0.01	+3.03	1.22	Yes
5	0.15	1	0.15 ± 0.01	0.15 ± 0.01	0.00	0.55	Yes
6	1.2	1	1.23 ± 0.01	1.23 ± 0.01	+2.49	0.67	Yes
7	22.1	1	22.15 ± 0.84	22.15 ± 0.84	+0.23	3.80	Yes
8	56.4	2	28.20 ± 0.45	56.40 ± 0.90	0.00	1.60	Yes
9	-	1	0.08 ± 0.002	<LOQ (detected)	-	2.10	No*
10	-	1	0.12 ± 0.002	<LOQ (detected)	-	1.80	No*
11-20	-	1	0.05-0.15	<LOQ (detected)	-	<5%	No*
21-40	Negative	1	Not detected	Not detected	-	-	No

*Below LOQ (0.357 µg/mL) — reported as semi-quantitative (detected but not accurately quantified)
*Antigen detected by immunoassay but culture-negative, suggesting non-viable bacteria or soluble antigen. For samples with concentrations below the LOQ (0.357 µg/mL) but above LOD (0.119 µg/mL), **"Detected (<LOQ)"** in the main manuscript. No %RE calculation (as these are semi-quantitative results)

Table S2: Determination of MRSA in farmer hand swab samples using the proposed Tb-2-Keton immunoassay compared with reference method.

Table S2: Determination of MRSA in Farmer Hand Swab Samples Using the Proposed Tb-2,Keton Immunoassay Compared with Reference Method

Sample No.	Reference Method (µg/mL)	Dilution Factor	Raw Measured (µg/mL)	Back-Calculated (µg/mL)	%RE	%RSD (n=3)	Confirmed Positive	Reporting Category
1	6.619	1	6.69 ± 0.09	6.69 ± 0.09	+1.09	1.28	Yes	Quantitative
2	0.065	1	0.066 ± 0.001	0.066 ± 0.001	—*	1.22	Yes	Below LOQ (detected)
3	0.017	1	0.0172 ± 0.0003	0.0172 ± 0.0003	—*	1.48	Yes	Below LOQ (detected)
4	0.212	1	0.2145 ± 0.003	0.2145 ± 0.003	—*	1.27	Yes	Below LOQ (detected)
5	3.778	1	3.82 ± 0.05	3.82 ± 0.05	+1.14	1.28	Yes	Quantitative
6	9.629	1	9.93 ± 0.22	9.93 ± 0.22	+3.16	2.23	Yes	Quantitative
7	21.35	1	21.26 ± 0.17	21.26 ± 0.17	-0.42	0.82	Yes	Quantitative
8	—	1	0.015 ± 0.0004	<LOQ (detected)	—*	2.50	No†	Below LOQ (detected)
9	—	1	0.021 ± 0.0006	<LOQ (detected)	—*	2.80	No†	Below LOQ (detected)
10	—	1	0.018 ± 0.0005	<LOQ (detected)	—*	2.60	No†	Below LOQ (detected)
11-40	Negative	1	Not detected	Not detected	—	—	No	Negative

Quantitative Results (Samples 1, 5, 6, 7): All measured values fall within the validated linear range of 3.0-26.8 µg/mL. %RE values are calculated based on back-calculated concentrations within the linear range. **Below LOQ (Samples 2, 3, 4, 8, 9, 10):** Samples with concentrations below the LOQ (0.357 µg/mL) but above the LOD (0.119 µg/mL) are reported as semi-quantitative ("detected <LOQ"). No %RE values are calculated for these samples as they fall outside the validated quantification range. **Statistical Justification for Below LOQ Reporting:** Samples 2, 3, and 4 were confirmed positive by the reference method (culture and PCR) but showed antigen concentrations below the LOQ. These are reported as "detected (<LOQ)" to indicate presence of MRSA antigen without claiming accurate quantification. This approach is consistent with IUPAC guidelines for reporting results below the LOQ. **†Antigen detected by immunoassay but culture-negative (Samples 8, 9, 10):** These samples show detectable antigen levels below the LOQ but were negative by culture and PCR, suggesting the presence of non-viable bacteria or soluble antigen not detectable by culture.

Table S3: Determination of MRSA in Cattle Drinking Water Samples Using the Proposed Tb-2,Keton Immunoassay Compared with Reference Method

Sample No.	Reference Method (µg/mL)	Dilution Factor	Raw Measured (µg/mL)	Back-Calculated (µg/mL)	%RE	%RSD (n=3)	Confirmed Positive	Reporting Category
1	3.65	1	3.68 ± 0.08	3.68 ± 0.08	+0.82	2.1	Yes	Quantitative
2	0.12	1	0.122 ± 0.003	0.122 ± 0.003	—*	2.8	Yes	Below LOQ (detected)
3	0.035	1	0.0345 ± 0.001	0.0345 ± 0.001	—*	3.2	Yes	Below LOQ (detected)
4	0.28	1	0.285 ± 0.007	0.285 ± 0.007	—*	2.5	Yes	Below LOQ (detected)
5	1.92	1	1.95 ± 0.04	1.95 ± 0.04	—*	2.2	Yes	Below LOQ (detected)
6	7.43	1	7.51 ± 0.14	7.51 ± 0.14	+1.08	1.8	Yes	Quantitative
7	0.068	1	0.069 ± 0.002	0.069 ± 0.002	—*	2.9	Yes	Below LOQ (detected)
8	0.51	1	0.515 ± 0.012	0.515 ± 0.012	—*	2.3	Yes	Below LOQ (detected)
9	2.36	1	2.39 ± 0.05	2.39 ± 0.05	—*	2.0	Yes	Below LOQ (detected)
10	15.82	1	15.96 ± 0.26	15.96 ± 0.26	+0.88	1.6	Yes	Quantitative
11	—	1	0.015 ± 0.0004	<LOQ (detected)	—*	2.8	No†	Below LOQ (detected)
12	—	1	0.025 ± 0.0007	<LOQ (detected)	—*	2.5	No†	Below LOQ (detected)
13-40	Negative	1	Not detected	Not detected	—	—	No	Negative

Quantitative Results (Samples 1, 6, 10): All measured values fall within the validated linear range of 3.0-26.8 µg/mL. %RE values are calculated based on back-calculated concentrations within the linear range. **Below LOQ (Samples 2, 3, 4, 5, 7, 8, 9, 11, 12):** Samples with concentrations below the LOQ (0.357 µg/mL) but above the LOD (0.119 µg/mL) are reported as semi-quantitative ("detected <LOQ"). No %RE values are calculated for these samples as they fall outside the validated quantification range. **Statistical Justification for Below LOQ Reporting:** Samples 2, 3, 4, 5, 7, 8, and 9 were confirmed positive by the reference method but showed antigen concentrations below the LOQ. These are reported as "detected (<LOQ)" to indicate presence of MRSA antigen without claiming accurate quantification. **†Antigen detected by immunoassay but culture-negative (Samples 11, 12):** These samples show detectable antigen levels below the LOQ but were negative by culture and PCR, suggesting the presence of non-viable bacteria or soluble antigen not detectable by culture.

Table S4: Recovery studies for MRSA antigen spiked into negative milk and hand swab samples (n=5).

Sample Matrix	Spiked Concentration (µg/mL)	Found Concentration (µg/mL)	Dilution factor	Recovery (%)	%RSD
Milk	5.0	4.93 ± 0.15		98.6	3.04
Milk	25.0	25.3 ± 0.62		101.2	2.45
Milk	100.0	12.725 ± 1.89	8	101.8	1.86
Hand Swab	1.0	0.97 ± 0.04		97.0	4.12
Hand Swab	10.0	10.2 ± 0.31		102.0	3.04
Hand Swab	50.0	8.23 ± 1.12	6	98.8	2.27

Table S5: Selectivity of the Tb-2,Keton immunoassay against potential interferents and cross-reacting species.

Interferent/Potential Cross-Reactant	Concentratio n	Fluorescence Response (F/F_0)	Relative Signal (%)*	Cross-Reactivity Category
Blank (PBS buffer)	-	1.00 ± 0.02	0	Negative
MRSA antigen	1.0 µg/mL	1.89 ± 0.03	100	Positive control
MSSA (heat-killed)	10^7 CFU/mL	1.08 ± 0.04	9.0	Low cross-reactivity
MRSE (heat-killed)	10^7 CFU/mL	1.09 ± 0.02	4.8	Negligible
MSSE (heat-killed)	10^7 CFU/mL	1.06 ± 0.02	3.2	Negligible
S. haemolyticus	10^7 CFU/mL	1.05 ± 0.02	2.9	Negligible
S. hominis	10^7 CFU/mL	1.04 ± 0.02	2.1	Negligible
E. faecium	10^7 CFU/mL	1.05 ± 0.02	3.1	Negligible
E. faecalis	10^7 CFU/mL	1.04 ± 0.02	2.4	Negligible
S. agalactiae	10^7 CFU/mL	1.03 ± 0.02	2.8	Negligible
B. subtilis	10^7 CFU/mL	1.03 ± 0.01	1.9	Negligible

*Relative signal = [(F/F_0 of interferent - 1)/(F/F_0 of MRSA - 1)] × 100

Table S6: Stability of Tb-2,Keton complex and immunoassay performance over time.

Storage Time (weeks)	Stored at 4°C			Stored at 25°C		
	F/F_0 ± SD (n=3)	% Initial Response	% RSD	F/F_0 ± SD (n=3)	% Initial Response	% RSD
0	5.42 ± 0.08	100	1.48	5.42 ± 0.08	100	1.48
1	5.39 ± 0.07	99.4	1.30	5.31 ± 0.09	98.0	1.69
2	5.35 ± 0.08	98.7	1.50	5.18 ± 0.10	95.6	1.93
3	5.29 ± 0.09	97.6	1.70	5.02 ± 0.11	92.6	2.19
4	5.21 ± 0.10	96.1	1.92	4.85 ± 0.12	89.5	2.47
6	5.08 ± 0.11	93.7	2.17	4.52 ± 0.14	83.4	3.10
8	4.93 ± 0.12	91.0	2.43	4.16 ± 0.15	76.8	3.61
10	4.74 ± 0.14	87.5	2.95	3.70 ± 0.16	68.2	4.32
12	4.56 ± 0.15	84.2	3.29	3.24 ± 0.17	59.7	5.25

(B) Degradation Kinetics Analysis:

Storage Time (weeks)	Tb-2,Keton Complex Stored at 4°C (% Initial Response)	Tb-2,Keton Complex Stored at 25°C (% Initial Response)
0	100	100
1	99.4	98.0
2	98.7	95.6
3	97.6	92.6
4	96.1	89.5
6	93.7	83.4
8	91.0	76.8
10	87.5	68.2
12	84.2	59.7

Table S7: Inter-batch reproducibility of Tb-2,Keton immunoassay.

MRSA Antigen Concentration (μg/mL)	Batch 1 (F/F_o)	Batch 2 (F/F_o)	Batch 3 (F/F_o)	Mean ± SD	%RSD
1.0	1.89	1.92	1.87	1.89 ± 0.03	1.37
5.0	2.43	2.48	2.41	2.44 ± 0.04	1.50
10.0	3.12	3.18	3.09	3.13 ± 0.05	1.48
25.0	4.28	4.35	4.24	4.29 ± 0.06	1.33
50.0	5.42	5.51	5.38	5.44 ± 0.07	1.26
100.0	6.85	6.98	6.81	6.88 ± 0.09	1.29

Table S8: Robustness testing of the Tb-2,Keton immunoassay for MRSA detection (50 µg/mL antigen).

Parameter	Variation	$F/F_0 \pm SD$	% Change from Optimal
Optimal conditions	-	5.42 ± 0.08	-
Excitation wavelength (nm)	318	5.38 ± 0.09	-0.7
	322	5.40 ± 0.07	-0.4
Emission wavelength (nm)	543	5.39 ± 0.08	-0.6
	547	5.41 ± 0.09	-0.2
pH of PBS buffer	7.2	5.38 ± 0.10	-0.7
	7.6	5.35 ± 0.11	-1.3
Incubation time (min)	4	5.37 ± 0.09	-0.9
	6	5.43 ± 0.08	+0.2
Temperature (°C)	23	5.40 ± 0.10	-0.4
	27	5.36 ± 0.12	-1.1
Tb-2,Keton concentration ($\times 10^{-4}$ M)	1.8	5.31 ± 0.13	-2.0
	2.2	5.45 ± 0.09	+0.6
